# Phytohormone sensing in the biotrophic fungus *Ustilago maydis* – the dual role of the transcription factor Rss1

**DOI:** 10.1111/mmi.13460

**Published:** 2016-08-08

**Authors:** Franziska Rabe, Denise Seitner, Lisa Bauer, Fernando Navarrete, Angelika Czedik‐Eysenberg, Fernando A. Rabanal, Armin Djamei

**Affiliations:** ^1^ Vienna Biocenter (VBC)Gregor Mendel Institute (GMI), Austrian Academy of Sciences (OEAW)Dr. Bohr‐Gasse 3Vienna1030Austria; ^2^ Present address: Department of Infectious Diseases & Immunology, Virology Division, Faculty of Veterinary MedicineUniversity of UtrechtYalelaan 1Utrecht3584CLNetherlands

## Abstract

The phenolic compound salicylic acid (SA) is a key signalling molecule regulating local and systemic plant defense responses, mainly against biotrophs. Many microbial organisms, including pathogens, share the ability to degrade SA. However, the mechanism by which they perceive SA is unknown. Here we show that *Ustilago maydis*, the causal agent of corn smut disease, employs a so far uncharacterized SA sensing mechanism. We identified and characterized the novel SA sensing regulator, Rss1, a binuclear zinc cluster protein with dual functions as putative SA receptor and transcriptional activator regulating genes important for SA and tryptophan degradation. Rss1 represents a major component in the identified SA sensing pathway during the fungus’ saprophytic stage. However, Rss1 does not have a detectable impact on virulence. The data presented in this work indicate that alternative or redundant sensing cascades exist that regulate the expression of SA‐responsive genes in *U. maydis* during its pathogenic development.

## Introduction

Salicylic acid (SA, 2‐hydroxybenzoic acid) belongs to the class of phenolic compounds and is composed of an aromatic ring with a carboxyl‐ and hydroxyl group. Its biosynthesis is shared between organisms of diverse kingdoms of life ranging from bacteria to plants (Serino *et al*., 1995; Kerbarh *et al*., 2005; Dempsey *et al*., 2011). In bacteria, SA serves as building block for siderophore biosynthesis or as siderophore itself (Visca *et al*., 1993). In plants, it was identified as important hormone regulating diverse processes, including seed germination, cell growth, respiration, and most prominently defense responses to biotrophic pathogens (Vlot *et al*., 2009). To limit the spread of biotrophic pathogens that rely on the living host for proliferation (Glazebrook, 2005), SA regulates a complex network of diverse signalling components. Upon pathogen attack, this network activates defense responses which can culminate in hypersensitive cell death at the site of infection (Vlot *et al*., 2009). Moreover, SA is involved in systemic acquired resistance in distal, non‐infected parts of the plant resulting in broad‐spectrum resistance to diverse pathogens and an immune memory that primes the plant for secondary infections (Fu and Dong, 2013).

One model pathogen for the study of biotrophic interactions between filamentous fungi and their hosts is the smut fungus *Ustilago maydis*. The basidiomycete overcomes SA‐regulated defense barriers of its host *Zea mays*, one of the most important crop plants, and successfully proliferates inside the plant (Doehlemann *et al*., 2008). After recognition during the early phase of infection, *U. maydis* suppresses the immune responses of its host with the help of small secreted molecules, termed effectors, and establishes a biotrophic interaction (Kamper *et al*., 2006; Doehlemann *et al*., 2008; Djamei *et al*., 2011; Hemetsberger *et al*., 2012; Mueller *et al*., 2013). The biotrophic interaction ultimately leads to tumor formation, where fungal proliferation and differentiation of black‐pigmented fungal spores occurs (Christensen, 1963). Not surprisingly, *U. maydis* as well as other (hemi‐) biotrophic plant pathogens employ diverse strategies to interfere with host SA signalling and production in order to suppress defense responses (Tanaka *et al*., 2015). For example, they can limit SA biosynthesis through the elimination of SA precursors, like chorismate or isochorismate (Djamei *et al*., 2011; Liu *et al*., 2014), or they manipulate the proteasome‐mediated turnover rate of SA signalling components (Ustun *et al*., 2013). Moreover, we recently showed that *U. maydis* degrades SA by means of the salicylate hydroxylase Shy1, demonstrating the capability of a biotrophic pathogen to eliminate SA (Rabe *et al*., 2013). Shy1 is essential for the utilization of SA as carbon source in axenic culture and the *shy1* gene is induced during plant colonization. However, since the deletion of *shy1* did not affect virulence, the role of SA‐degradation in the pathogenic development of *U. maydis* remains elusive. Besides Shy1, two additional proteins were predicted to be salicylate hydroxylases but did not show enzymatic activity. Respective genes are upregulated during pathogenic development and show an increase in transcript levels upon SA treatment. The specific induction of a set of genes in the presence of SA indicates that *U. maydis* is able to sense SA by an as yet unknown mechanism. Shy1 is thought to be part of a negative feedback loop, indirectly regulating SA‐responsive gene expression. By degrading SA, Shy1 reduces the amount of inducer, which subsequently leads to the downregulation of SA‐responsive genes (Rabe *et al*., 2013).

Studies of model plants like Arabidopsis and tobacco have provided insights into SA perception and signalling in plants, and several SA receptors and binding proteins have been discovered in the recent years (Seyfferth and Tsuda, 2014). Although SA binding is well studied, divergent models propose different bona fide SA receptors important for regulating pathogen defense. Wu and colleagues (2012) showed that upon SA binding the transcriptional co‐activator NPR1 changes its conformation and activates defense‐related genes. Fu *et al*. (2012) failed to show SA binding by NPR1 and propose NPR3 and NPR4 as bona fide SA receptors. Both proteins bind SA and, depending on the cellular SA concentration, target NPR1 for proteasomal degradation. Moreover, H_2_O_2_ scavengers and methyl salicylate esterases are capable of binding SA with high affinities resulting in inhibition of their enzymatic activity (Chen and Klessig, 1991; Durner and Klessig, 1995; Kumar and Klessig, 2003; Forouhar *et al*., 2005).

Since many filamentous fungi have evolved ways to eliminate SA (Sze and Dagley, 1984; Penn and Daniel, 2013; Rabe *et al*., 2013; Ambrose *et al*., 2015; Martins *et al*., 2015), SA sensing should also be wide spread among these organisms. However, nothing is known about fungal SA perception and signalling. Here we show that the biotrophic fungus *U. maydis* perceives SA via the response factor Rss1, a putative binuclear zinc cluster protein. The protein constitutes a major component of SA sensing and regulates genes involved in a shared pathway for the metabolism of SA and tryptophan. However, although Rss1 is essential for the activation of SA‐responsive genes in the saprophytic phase of *U. maydis*, we provide evidence that additional cues and pathways exist that regulate these genes during plant colonization.

## Results

### Isolation of *UMAG_05966* as an important factor for SA sensing

**Figure 1:**
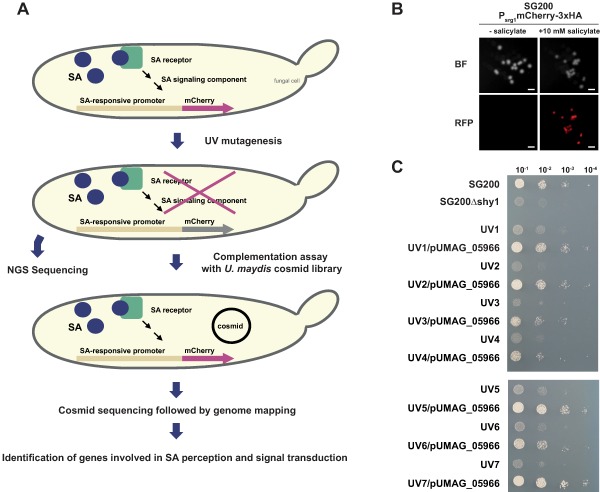
UMAG_05966 (Rss1) represents a major component of SA perception and signalling in *U. maydis*. A. Schematic representation of the UV mutagenesis screen to identify proteins involved in SA sensing. SG200P_srg1_mCherry‐3xHA, expressing *mCherry‐3xHA* under control of the SA‐responsive *srg1* promoter, was mutagenized with 20 mJ UV. Cells were screened for loss of fluorescence, i. e. for inability to activate the *srg1* promoter. Candidate mutants were subjected to NGS whole genome sequencing and complementation analysis employing an autonomously replicating *U. maydis* cosmid library (Weinzierl, 2001). Re‐isolated cosmids from rescue mutants were sequenced and mapped to the *U. maydis* genome. Results were compared to NGS sequencing data. B. Epifluorescence stereomicroscopy was performed with SG200P_srg1_mCherry‐3xHA colonies on glucose‐containing YNB‐N medium without (left panel) and with 10 mM sodium salicylate (right panel). Fluorescence could only be detected for SG200P_srg1_mCherry‐3xHA spotted on salicylate‐containing medium, indicating a specific and strong activation of the chosen SA‐responsive promoter. Scale bars: 1 mm. C. UV mutant strains with loss of fluorescence were spotted on YNB‐N + 10 mM salicylate and exhibited severe growth retardation similar to SG200Δshy1. Growth retardation was rescued after introduction of pUMAG_05966 expressing *UMAG_05966* under the endogenous *UMAG_05966* promoter.

We have previously shown that *U. maydis* senses SA resulting in the transcriptional induction of SA‐responsive genes. Among these are the salicylate hydroxylase encoding gene *shy1*, essential for SA degradation, as well as the SA induced gene *srg1* (*SA‐responsive gene 1*; *UMAG_05967*), coding for a protein of so far unknown function (Rabe *et al*., 2013). To identify factors involved in the SA sensing pathway we conducted a genetic screen by making use of the strong *srg1* promoter. The haploid solopathogenic strain SG200 was transformed with a construct harbouring *mCherry‐3xHA* under control of the *srg1* promoter and subjected to UV mutagenesis to retrieve mutants impaired in SA perception and signalling (Fig. 1A). Since SG200P_srg1_mCherry‐3xHA exhibited strong and specific mCherry fluorescence in the presence of SA (Fig. 1B), loss of fluorescence was used as a readout after UV mutagenesis. If mutations led to the disruption of the SA sensing pathway, the respective strains should be unable to activate the *srg1* promoter and to produce mCherry‐3xHA upon SA‐treatment. Seven of 86,400 tested colonies showed the expected phenotype and did not exhibit mCherry fluorescence (Supporting Information Fig. 1A). Since induction of *shy1* is essential for SA degradation under axenic growth conditions, a disrupted SA sensing pathway might lead to reduced *shy1* gene expression and impaired growth on medium with SA as sole carbon source. In line with this assumption, all identified mutants displayed severe growth attenuations on salicylate minimal medium (Fig. 1C). To identify the mutations underlying the phenotype, each mutant strain was transformed with an *U. maydis* cosmid library (Weinzierl, 2001) and screened for rescue of the mutation through restored mCherry fluorescence. Four candidate genes (*UMAG_10865*, *UMAG_05964*, *UMAG_05965*, *UMAG_05966*) located at the end of chromosome 20 were identified as putatively involved in SA sensing. Complementation analysis with autonomously replicating plasmids harbouring the individual genes revealed that *UMAG_05966* was able to rescue the SA sensing phenotype of all mutants. *UMAG_05966* complemented mutant strains were able to grow on minimal medium with salicylate as sole carbon source (Fig. 1C) and showed restored mCherry fluorescence (Supporting Information Fig. 1B). Since *UMAG_05966* is located on an autonomously replicating plasmid, which is present in the cell in multiple copies (Tsukuda *et al*., 1988), its overexpression might have rescued other mutations underlying the mutant phenotype. To exclude this, Next Generation Illumina Sequencing (NGS) and single nucleotide polymorphism (SNP) analysis were performed. Although we found several unique mutations for each mutant in different genomic regions (average number of mutations per genome = 12 ± 9; Supporting Information Table 1), only the chromosomal region encompassing *UMAG_05966* harboured mutations or deletions in all sequenced strains: *UMAG_05966* of UV1 and UV6 contained non‐synonymous mutations while all other mutants lost more than 45 kb from the end of chromosome 20 including *UMAG_05966* (Supporting Information Fig. 2). This makes it unlikely, that additional mutations led to the observed phenotype. Through targeted gene deletion of *UMAG_05966* via homologous recombination we confirmed the observed growth defect on SA minimal medium indicating that the deletion of *UMAG_05966* blocks SA perception and/or signalling (Supporting Information Fig. 3). The growth attenuation could be partially complemented by ectopic expression of *mCherryHA‐UMAG_05966* under control of the *UMAG_05966* promoter and fully rescued by an untagged version of *UMAG_05966* (Supporting Information Fig. 3). Based on the NGS and complementation data we concluded that UMAG_05966 is a key player in SA perception and/or signal transduction under the tested conditions, and we designated the protein Rss1 (Required for SA sensing 1).

### Rss1 likely has a dual function as transcriptional activator and putative SA receptor

**Figure 2:**
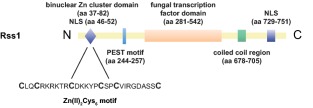
Rss1 harbours domains of binuclear zinc cluster transcription factors. Rss1 (UMAG_05966) contains domains and sequences known to be present in binuclear zinc cluster transcription factors including predicted Nuclear Localization Signal (NLS) sequences, a Zn(II)_2_Cys_6_‐DNA binding domain, a putative fungal transcription factor domain, a predicted PEST motif for proteasomal turnover and a coiled‐coil domain important for dimerization. Predicted domains are indicated.

*rss1* is located in the *U. maydis* genome on chromosome 20, upstream of the SA‐responsive gene *srg1*. Both genes share the same promoter region and they are divergently transcribed (A. Czedik‐Eysenberg, J. Bindics, unpublished tiling array data). *rss1* encodes an 866 amino acid (aa) long protein that harbours domains often found in ligand binding binuclear zinc cluster transcription factors (Fig. 2; MacPherson *et al*., 2006; Shelest, 2008): a putative N‐terminal Zn(II)_2_Cys_6_‐DNA binding domain (aa 37–82), a predicted PEST motif (aa 244‐257) known to promote proteasomal degradation, a putative fungal transcription factor region (aa 281‐542), a coiled coil region considered to be important for dimerization (aa 678–705), and a predicted monopartite nuclear localization signal (NLS) at the N‐ and a bipartite NLS at the C‐terminus (aa 46–52 and 729–751; Nucpred = 0.95). The consensus sequence CX_2_CX_6_CX_5‐9_CX_2_CX_6‐8_C of the DNA binding domain, which is characteristic for binuclear zinc cluster proteins (MacPherson *et al*., 2006), is highly conserved in Rss1 (Fig. 2).

**Figure 3:**
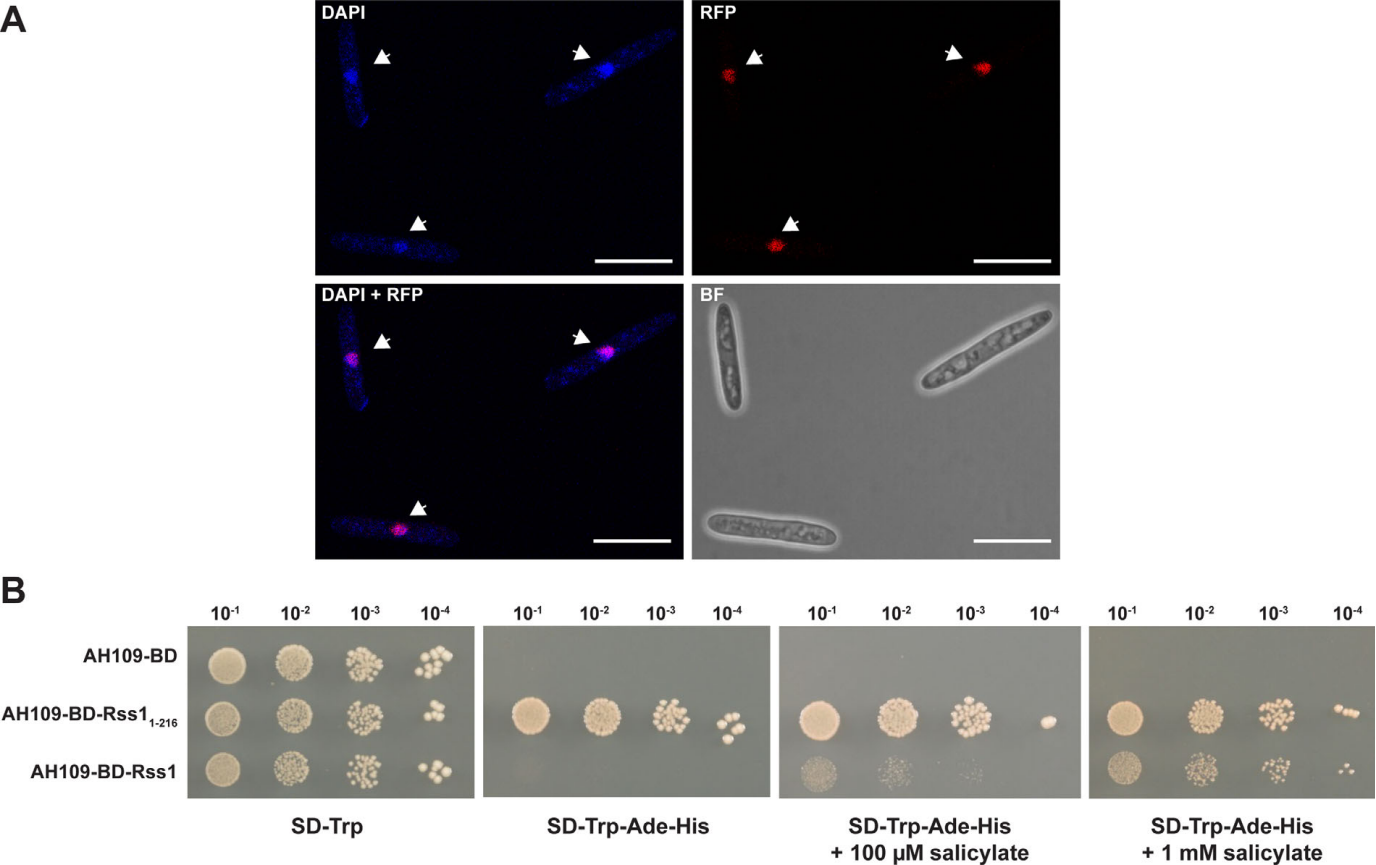
Rss1 localizes to *U. maydis* nuclei and responds to SA as a transcriptional activator in a heterologous system. A. mCherryHA‐Rss1 localizes to the nuclei of *U. maydis* yeast‐like cells. Localization of mCherryHA‐Rss1 in cells of axenically grown CL13Δrss1‐mCherryHA‐rss1 culture was assessed by confocal laser scanning microscopy. Cells were stained with DAPI to visualize nuclei. Scale bars: 10 μm. B. Gal4‐BD‐Rss1 activates reporter gene expression in *S. cerevisiae* in the presence of salicylate and shows a dose‐dependent response to SA. AH109 expressing *Gal4‐BD* (AH109‐BD; negative control), *Gal4‐BD‐rss1_1‐216_* (AH109‐BD‐Rss1_1‐216_; positive control), or *Gal4‐BD‐rss1* (AH109‐BD‐Rss1) was spotted in serial dilutions on SD‐Trp, SD‐Trp‐Ade‐His, SD‐Trp‐Ade‐His + 100 µM sodium salicylate and SD‐Trp‐Ade‐His + 1 mM sodium salicylate. Under stringent conditions growth of AH109‐BD‐Rss1 was only detectable with addition of salicylate.

In line with its predicted function as a transcription factor and its nuclear localization signals, mCherryHA‐Rss1 exclusively localizes to nuclei of *U. maydis* cells grown in YNB‐N supplemented with 10 mM SA (Fig. 3A). Differences in localization in SA‐treated and untreated cells could not be observed (data not shown).

Since bioinformatic predictions as well as the confirmed nuclear localization supported a function as transcription factor, we tested whether Rss1 acts as transcriptional activator. To this end, the DNA binding domain of the Gal4 transcription factor of *Saccharomyces cerevisiae* (Gal4‐BD) was fused to the N‐terminus of Rss1. The respective construct was introduced into *S. cerevisiae* AH109 harbouring auxotrophy markers for histidine and adenine biosynthesis that are under control of Gal4‐responsive promoters. Due to recruitment of the fusion protein to the Gal4‐responsive promoters by Gal4‐BD, the capability of Rss1 to act as transcriptional activator could be monitored by the induction of reporter gene expression. Transcriptional induction eventually leads to growth on high stringency medium. The Gal4‐BD‐Rss1 producing strain AH109‐BD‐Rss1 did not grow on high stringency medium (Fig. 3B). However, the addition of 100 µM or 1 mM salicylate restored growth of AH109‐BD‐Rss1 in a dose‐dependent manner (Fig. 3B). We excluded that the exclusively SA‐dependent induction represents an artifact of the medium by including AH109‐BD, expressing Gal4‐BD, as negative control and a strain producing a truncated autoactive mutant version of Rss1, Rss1_1‐216_, fused to Gal4‐BD as positive control.

Since we could not find any Rss1 orthologs in *S. cerevisiae* by BlastP analysis and the strain used in this study was unable to utilize SA as carbon source (Supporting Information Fig. 4), it is unlikely that an orthologous SA sensing cascade in yeast activates Rss1 post‐translationally. Therefore, we reasoned that Rss1 might not only represent a transcription factor but at the same time a receptor for SA. To test SA binding, we tried to produce Rss1 with N‐terminal affinity tag fusions either in *U. maydis* or heterologously in *Escherichia coli*. However, since all tested production methods resulted in protein aggregation, no, or highly unstable soluble protein (Supporting Information Table 2), we could not directly determine SA binding by Rss1 and its binding affinity.

Dimerization of binuclear zinc cluster proteins is often necessary to bind DNA and to regulate transcription (MacPherson *et al*., 2006). By employing the Yeast two‐hybrid system we could show that Rss1 forms homodimers (Supporting Information Fig. 5). In addition, the presence of the binuclear zinc cluster domain suggests, that Rss1 directly recognizes and binds a certain motif present in its target promoters, e.g. in the *srg1* promoter region. However, since the production of biologically active Rss1 protein failed, we could not assess DNA binding and identify specific Rss1‐binding motifs.

### *rss1* is transcriptionally induced during pathogenic development but does not have a detectable impact on virulence

**Figure 4:**
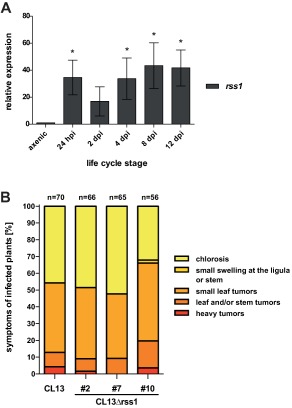
*rss1* is transcriptionally induced during the pathogenic development of *U. maydis* but has no impact on virulence. A. Transcript levels of *rss1* were determined by quantitative real time PCR after RNA isolation and cDNA synthesis from SG200 axenic culture and from different developmental stages. Constitutively expressed *peptidyl‐prolyl isomerase* (*ppi*) was used for normalization. Transcript levels were compared to those in axenic culture and levels in axenic culture were set to 1.0. Error bars depict standard deviation calculated from three independent biological replicates using infected areas from 12 plants each (*n* = 3). Significance was calculated with unpaired *t* test comparing expression values with those of axenic culture, * *P* ≤ 0.05. B. Disease symptoms of maize seedlings were determined 12 days post infection with either *U*. *maydis* CL13 or three independent isolates of CL13Δrss1 according to Kamper *et al*. (2006). Disease symptom categories are colour‐coded and depicted on the right. Mean values from two independent infections are shown with the total number of infected plants above each column. No significant differences in disease symptoms were scored.

Since SA‐responsive genes, like *shy1* and *srg1*, are transcriptionally induced during pathogenic development of *U. maydis*, we reasoned that their regulator might also be present during those developmental stages. To test whether *rss1* is expressed, we monitored *rss1* transcript levels at different infection stages of the solopathogenic strain SG200 by quantitative real time PCR. *rss1* expression was induced 35‐fold in the early stage of infection compared to axenic culture, decreased slightly at two days post infection (dpi), but increased again at four dpi. Transcript levels stayed elevated up to 43‐fold until sporogenesis occurred at twelve dpi (Fig. 4A). Since genes involved in pathogenic development are often induced upon plant infection (Kamper *et al*., 2006), the expression profile of *rss1* suggested that the protein might play a role in virulence. To investigate whether SA sensing by Rss1 is important for the pathogenic development of the fungus, virulence assays with CL13Δrss1 and the progenitor strain CL13 were performed. The haploid solopathogenic strain CL13 was chosen for this experiment, since modest virulence defects can be better assessed with this strain than with SG200 (Di Stasio *et al*., 2009; Djamei *et al*., 2011). However, *rss1* deletion strains showed no significant alteration in virulence in seedling infections (Fig. 4B). This indicates either that SA perception and signalling are not relevant for virulence under standard laboratory conditions or that Rss1‐mediated SA sensing is part of a complex network where the lack of one pathway can be compensated by others.

### Rss1‐independent SA sensing pathways exist regulating SA‐responsive genes during pathogenic development

**Figure 5:**
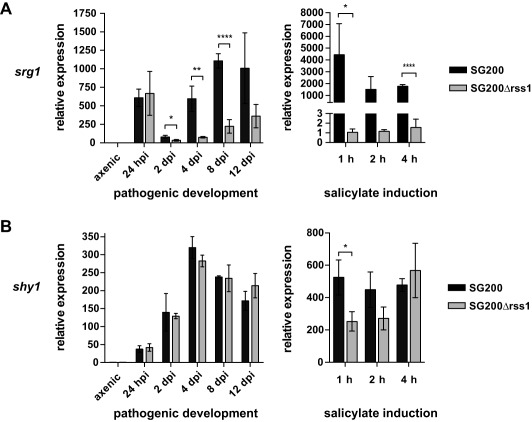
Transcriptional induction of SA‐responsive genes during pathogenic development and during growth in SA supplemented medium does not exclusively depend on Rss1. Transcript levels of the previously identified SA‐responsive genes *srg1* (A) and *shy1* (B) were quantified in SG200 and SG200Δrss1 by real time PCR. RNA was isolated from the indicated life cycle stages of pathogenic development (‘pathogenic development’, left panel) and from a time course after shift to YNB‐N medium containing 2% glucose and 10 mM salicylate (‘salicylate induction’, right panel). Constitutively expressed peptidyl‐prolyl isomerase transcript levels (*ppi*) were used for normalization. Transcript levels of the indicated genes were either compared to levels in axenic culture grown in YEPS_light_ medium (left panel) or grown in YNB‐N medium with 2% glucose (right panel). Expression levels in axenic culture (left panel) or in cultures grown in YNB‐N with glucose (right panel) were set to 1.0. Error bars depict standard deviation calculated from three independent biological replicates (*n* = 3). Significance was calculated with unpaired *t* test comparing transcript levels of indicated genes in SG200 with those in SG200Δrss1, * *P* ≤ 0.05, ** *P* ≤ 0.01, *** *P* ≤ 0.001. For transcriptional profiling of different life cycle stages, RNA extracted from twelve infected plants per time point and replicate was used.

To investigate whether compensatory SA sensing pathways are active during plant infection, we determined the expression of previously identified SA‐responsive genes (Rabe *et al*., 2013) in SG200Δrss1 and its progenitor SG200 during pathogenic development and in a time course of SA‐treated axenic culture (Fig. 5). During pathogenic development *srg1* was significantly upregulated in SG200 compared to axenic culture (*P* < 0.022). SG200Δrss1 failed to induce *srg1* expression to the same degree as SG200 resulting in up to 8‐fold lower transcript levels. However, *srg1* expression in SG200Δrss1 was still significantly up‐regulated during pathogenic development (*P* < 0.018) and *srg1* transcript levels increased over time with a peak of 360‐fold at 12 dpi compared to axenic culture (Fig. 5A, left panel). A different expression pattern was observed when measuring *srg1* transcript levels in SA‐treated axenic cultures. While *srg1* transcript levels were induced up to 4,400‐fold in SG200, no significant induction of *srg1* could be detected in the Δ*rss1* mutant strain. Expression of *srg1* in SG200Δrss1 was reduced more than 4,000‐fold compared to SG200 (Fig. 5A, right panel). These findings indicate that, in contrast to axenic culture, Rss1‐independent pathways must exist that activate *srg1* expression *in planta*.

Surprisingly, *shy1* expression was not significantly altered during pathogenic development of SG200Δrss1 compared with SG200 (Fig. 5B, left panel). Upon salicylate treatment of axenic culture, *shy1* transcript levels were initially 2‐fold reduced in the *rss1* mutant compared to the progenitor strain but levels increased again over time (Fig. 5B, right panel).

In line with the finding that additional pathways contribute to the regulation of SA‐responsive genes, global transcriptional profiling of SG200 and SG200Δrss1 identified only nine genes that were differentially regulated during plant infection at four dpi (adjusted *P*‐value < 0.05). Six of those were significantly repressed in SG200Δrss1 while three were induced (Table 1). The majority of them showed only minor differences in expression levels compared to SG200 (fold change < 2.0; Table 1). Among the repressed genes were *srg1*, the SA marker gene, as well as *UMAG_02142* and *UMAG_12178*, which encode enzymes predicted to be involved in fungal metabolic pathways. UMAG_02142 displays homologies to muconate cycloisomerases, which are known to act in degradation pathways downstream of catechol (Martins *et al*., 2015). The functional classification system FunCat 2 (Ruepp *et al*., 2004) predicts that *UMAG_12178*, the gene with the most severe reduction in transcript levels (Log FC = −2.069), is involved in the metabolism of secondary products derived from tryptophan. Among the induced genes, *UMAG_06076* codes for a putative sugar transporter specialized in quinate uptake. In line with the expression data based on quantitative real time PCR, *shy1* was not among the differentially regulated genes. Since *UMAG_02142* might be involved in the downstream degradation of SA, we assessed the transcriptional profile of this gene by quantitative real time PCR under different conditions: *UMAG_02142* was transcriptionally induced upon SA treatment of axenic culture in SG200 and during pathogenic development. Similar to *srg1*, its transcript levels were significantly reduced in SG200Δrss1 (*P* < 0.019) and the reduction was more severe in axenic culture one hour after SA‐shift than during biotrophic growth (Supporting Information Fig. 6).

**Table 1:** Differentially expressed genes in SG200Δrss1 compared to SG200 at 4 dpi.

Gene ID	Log FC	*P*‐value	Adjusted *P*‐value	Gene prediction
*UMAG_12178*	−2.069	3.23 E −26	0.00000	Related to o‐pyrocatechuate decarboxylase
*UMAG_02142*	−0.805	5.49 E −09	0.00002	Related to muconate cycloisomerase
*UMAG_11117*	−0.58	1.99 E −05	0.01930	Uncharacterized protein
*UMAG_11020*	−0.551	3.09 E −05	0.02620	Uncharacterized protein
*UMAG_04481*	−0.533	4.75 E −05	0.03580	Related to ADH2 ‐ Alcohol dehydrogenase II
*UMAG_05967*	−0.542	6.90 E −05	0.04680	SA‐responsive gene *srg1*
*UMAG_03349*	0.625	1.69 E −05	0.01920	Uncharacterized protein
*UMAG_06076*	0.672	1.45 E −05	0.01920	Related to quinate transport protein
*UMAG_05819*	0.611	6.33 E −06	0.01070	Uncharacterized protein

### Rss1 is essential for utilizing tryptophan as a carbon source

**Figure 6:**
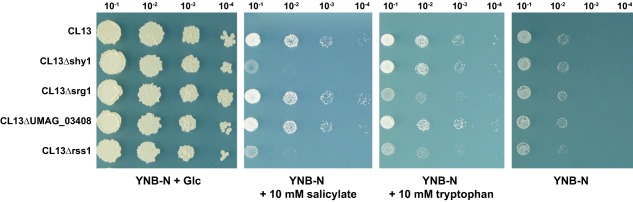
CL13Δrss1 and CL13Δsrg1 show growth attenuation on medium with tryptophan as sole carbon source. Growth of CL13 and deletion mutants of the SA‐responsive genes *shy1*, *srg1*, and *UMAG_03408* as well as CL13Δrss1 was assessed on YNB‐N supplemented with 2% glucose (YNB‐N + Glc), with 10 mM sodium salicylate (YNB‐N + 10 mM salicylate), with 10 mM tryptophan (YNB‐N + 10 mM tryptophan), or without any carbon source (YNB‐N). While *shy1* and *UMAG_03408* were not required for growth on tryptophan as sole carbon source, deletion of *srg1* and *rss1* resulted in growth attenuation on the respective medium. Images for ‘YNB‐N + Glc’ plate were acquired three days after spotting, for ‘YNB‐N + 10 mM salicylate’ plate after four days, and for ‘YNB‐N + 10 mM tryptophan’ and ‘YNB’ plates after six days.

Since global transcriptional profiling data indicated that Rss1 does not only regulate genes of the downstream pathway of catechol but might also be involved in the regulation of genes for tryptophan degradation, we assessed whether *U. maydis* is impaired in growth on tryptophan minimal medium in absence of *rss1*. Indeed, *rss1* deletion mutants showed attenuated growth when tryptophan was provided as sole carbon source (Fig. 6). Similar to the growth attenuation of CL13Δrss1, the deletion of *srg1* also resulted in growth retardation on tryptophan minimal medium (Fig. 6). To test whether tryptophan is an inducer of Rss1 activity, we repeated the heterologous yeast‐based transcriptional activation assay with tryptophan. In contrast to medium supplemented with salicylate, AH109‐BD‐Rss1 failed to grow when tryptophan was added (Supporting Information Fig. 7). These results indicate that Rss1 might not perceive tryptophan as a direct signal leading to its activation. The inability of Rss1 to sense tryptophan is also reflected by transcriptional profiling of SA‐responsive genes. Expression levels of the SA‐responsive genes *shy1*, *srg1*, and *UMAG_02142* were quantified by real time PCR and compared to those in untreated control cells. All tested genes showed significant lower transcript levels upon tryptophan treatment than after addition of salicylate (*P* < 0.033): *shy1* and *UMAG_02142* were only 2‐ and 6‐ fold induced upon tryptophan treatment compared to 388‐ and 34‐fold induction upon salicylate treatment (Supporting Information Fig. 8). *srg1* showed the highest induction (550‐fold) after the shift to tryptophan‐containing medium. However, the induction was significantly lower than after growth in medium supplemented with salicylate (*P* = 0.033), which resulted in a relative expression of more than 1,800‐fold (Supporting Information Fig. 8). The significantly weaker induction of SA‐responsive genes upon tryptophan treatment together with the transcriptional activation assay suggests that secondary products derived from the amino acid, and not tryptophan itself, are probably capable of activating the expression of the tested genes.

### Anthranilic acid, a possible degradation product of tryptophan, can modulate Rss1 activity

**Figure 7:**
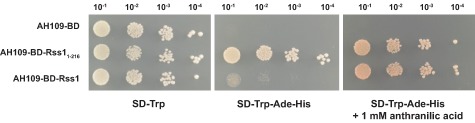
Anthranilate can induce Rss1 activity in yeast. The yeast‐based transcriptional activation assay was performed with anthranilate as a putative inducer. AH109 expressing *Gal4‐BD* (AH109‐BD; negative control), *Gal4‐BD‐rss1_1‐216_* (AH109‐BD‐Rss1_1‐216_; positive control), or *Gal4‐BD‐rss1* (AH109‐BD‐Rss1) were spotted in serial dilutions on SD‐Trp (growth control) (A), on SD‐Trp‐Ade‐His (B) and on SD‐Trp‐Ade‐His + 1 mM anthranilic acid (C). Addition of anthranilic acid resulted in an activation of reporter gene expression and growth of AH109‐BD‐Rss1.

Tryptophan degradation via L‐kynurenine is widespread among eukaryotes and is found in both fungi and animals (Ternes and Schonknecht, 2014). For fungi, it was shown that L‐kynurenine is converted into anthranilate which then is channeled via several intermediates into the 3‐oxoadipate pathway (Rao *et al*., 1971; Anderson and Dagley, 1981; Martins *et al*., 2015). SA and anthranilate are structurally similar, differing only in one of their two functional groups. Therefore, we tested whether anthranilate can activate Rss1 by repeating the yeast‐based transcriptional activation assay with anthranilate as a putative inducer. The addition of anthranilate resulted in growth on high stringency medium, suggesting that Rss1 is able to sense this compound and to activate reporter gene expression (Fig. 7).

### SA sensing is conserved among smuts

Since the smuts *Sporisorium reilianum* and *Ustilago hordei* share highly conserved orthologs of SA‐responsive genes with *U. maydis* (Rabe *et al*., 2013), their capability to respond to SA with an induction of those genes was tested by quantitative real time PCR. To this end, *S. reilianum* SRZ1 and *U. hordei* Uh4875‐4 were shifted to YNB‐N medium supplemented with glucose and 10 mM salicylate for one hour. Upon salicylate treatment the *shy1* ortholog in *S. reilianum*, *sr_shy1*, was only weakly induced, while *U. hordei* responded to SA with a 150‐fold transcriptional induction of *UHOR_shy1*. For *srg1* orthologs a different expression pattern was observed: *sr_srg1* in *S. reilianum* showed more than 100‐fold higher transcript levels upon SA treatment compared to levels in untreated cells, whereas *UHOR_srg1* was not significantly induced in an identical experimental set‐up (Supporting Information Fig. 9). The transcriptional profiles indicate that, although transcript levels of orthologous genes vary dramatically between smuts, SA can trigger the induction of genes in species related to *U. maydis*.

BlastP analyses revealed highly conserved Rss1 orthologs in *S. reilianum* (Sr16594), *Sporisorium scitamineum* (SPSC_06050), and *Melanopsichium pennsylvanicum* (Bn887_02897; Supporting Information Fig. 10). Rss1 and its orthologs in *S. reilianum* and *S. scitamineum* show conserved local synteny and orientation. Although *U. hordei* shares synteny of the respective region, no *rss1* ortholog could be found in the fungal genome. The presence of small blocks of *rss1* coding sequence remaining in the syntenic region of *U. hordei* indicates that the fungus likely once harboured a functional ortholog but might lost it after divergence from *U. maydis*.

## Discussion

In this study, we provide insights into a novel SA sensing and degradation mechanism in *U. maydis*. We show that a biotrophic fungus is able to sense SA by means of the response factor Rss1. This multifunctional protein, belonging to the family of binuclear zinc cluster proteins, constitutes a major component of an SA sensing mechanism present in *U. maydis*. It perceives SA and the structural analog anthranilate, presumably by direct binding, and regulates the expression of genes for SA and tryptophan degradation. SA signal transduction by Rss1 represents a novel mechanism that has not been described in other organisms. Rss1 has no homology to known SA sensing and signalling regulators in plants, such as the NPR proteins, thus making an identical mode of action unlikely (Fu *et al*., 2012; Wu *et al*., 2012). Also, bioinformatic comparisons with the bacterial SA response factor NahR did not reveal any significant similarity (data not shown) (Schell and Poser, 1989; Schell *et al*., 1990) suggesting that sensing and activation by Rss1 differs from bacterial systems and may have evolved independently.

Rss1 harbours domains known for binuclear zinc cluster transcription factors (Fig. 2). Binuclear zinc cluster proteins are exclusively found in fungi and they regulate diverse cellular processes, including sugar and amino acid metabolism, nitrogen utilization, respiration, as well as cell cycle regulation (MacPherson *et al*., 2006; Shelest, 2008). Moreover, they often share functional similarity with transcription factors of the nuclear receptor protein family found in metazoans. Nuclear receptor proteins are able to bind small‐molecule ligands and, as a result of ligand binding, activate the transcription of target genes (Naar and Thakur, 2009). The bioinformatic predictions (Fig. 2) together with the data of the yeast‐based transcriptional activation assay (Fig. 3) suggest that Rss1 might share the ability to act both as signalling sensor and concomitantly as a transcriptional activator. The binuclear zinc cluster proteins Leu3p and Pdr1 of *S. cerevisiae* are, like many other proteins of this class, composed of an internal domain with low conservation, termed the middle homology region (MHR) (MacPherson *et al*., 2006). For Pdr1, the MHR domain is important for ligand binding. It was shown that ligand binding leads to a conformational change of Pdr1 which in turn enables the C‐terminal activation domain to interact with a subunit of the Mediator complex and to initiate transcription of target genes (Thakur *et al*., 2008). Moreover, mutations in this region render the protein constitutively active (Carvajal *et al*., 1997; Nourani *et al*., 1997). Similar to Pdr1, deletion and mutational analyses of Rss1 as along with interaction studies might provide further insight into which domains are important for ligand binding and how Rss1 triggers transcriptional activation.

Several binuclear zinc cluster proteins, including the Pdr1 paralog Pdr3, were shown to form positive autoregulatory feedback loops modulating their own expression (Delahodde *et al*., 1995; Zhang *et al*., 2001). Since *rss1* transcript levels are significantly upregulated during pathogenic development (Fig. 4) and *rss1* shares the promoter region with its target gene *srg1*, it is conceivable that Rss1 also regulates its own expression.

Due to their coiled coil domains binuclear zinc cluster proteins often form homo‐ or heterodimers that recognize response elements with CGG triplets as inverted, everted, or direct repeats (Hellauer *et al*., 1996; Todd and Andrianopoulos, 1997; MacPherson *et al*., 2006). In line with this, Rss1 forms a homodimer (Supporting Information Fig. 5) and its promoter as well as those of its putative target genes, like *srg1*, *UMAG_02142* and *UMAG_01278*, contain several inverted and direct CGG repeats. It will be of future interest to experimentally elucidate the binding motif of the Rss1‐homodimer.

Although Rss1 is an essential factor in SA sensing in the saprophytic phase of the fungus, virulence was not altered when *rss1* was deleted (Fig. 4). Since we showed that transcript levels of target genes are more dramatically reduced in axenic culture than during pathogenic development in the *rss1* mutant (Fig. 5, Supporting Information Fig. 6), we conclude that alternative signalling pathways must be present during biotrophic growth. However, the identification of pathways that are exclusively active in biotrophy will be technically challenging.

SA growth assays presented in this work show that Rss1 regulates genes involved in SA degradation (Supporting Information Fig. 3). We supported these findings by global transcriptional profiling (Table 1). Among Rss1‐regulated targets are genes that may be involved in the downstream metabolism of SA, such as the putative muconate cycloisomerase encoding gene *UMAG_02142*. However, the SA sensing and degradation pathway we identified seems to be tightly linked to tryptophan metabolism and Rss1 represents a regulator for the metabolism of both compounds. Since Srg1, although displaying sequence similarity to salicylate hydroxylases, is not involved in the conversion of SA to catechol (Rabe *et al*., 2013) but is essential for tryptophan degradation (Fig. 6), we assume that the protein converts the structural analog of SA, anthranilate. The intermediate 2,3‐dihydroxybenzoate, which is produced by anthranilate hydroxylases (Rao *et al*., 1971), could be subsequently converted to catechol by the Rss1 target UMAG_12178. UMAG_12178 is related to o‐pyrocatechuate decarboxylases which facilitate the aforementioned reaction (Rao *et al*., 1967). Catechol can be further metabolized via the 3‐oxoadipate pathway and TCA cycle (Martins *et al*., 2015). Moreover, we provide evidence that by reacting to the tryptophan degradation product anthranilate Rss1 could be directly involved in the regulation of this pathway (Fig. 7). In contrast to *srg1*, the deletion of *shy1* had no impact on growth on tryptophan as sole carbon source (Fig. 6). The production of catechol by salicylate hydroxylases (Yamamoto *et al*., 1965) makes it likely that the Shy1‐mediated SA degradation pathway converges with the tryptophan branch and the shared intermediate catechol enters the 3‐oxoadipate pathway. Although *shy1* was not differentially regulated in a *rss1* deletion mutant during pathogenic development, transcript levels were significantly reduced in the early phase after shifting cells to SA containing medium (Fig. 5), indicating that Rss1 contributes, albeit only under certain conditions, to the regulation of *shy1* and SA degradation. Whether the ability to sense SA and regulate *shy1* expression represents a neofunctionalization of Rss1 or is an evolutionary remnant which will disappear over time remains speculative.

It might not be coincidental that genes involved in tryptophan metabolism are induced during biotrophy. Many phytopathogenic fungi are able to metabolize amino acids as carbon and nitrogen sources (Solomon *et al*., 2003). Among these are obligate biotrophic fungi, like *Blumeria graminis* and *Uromyces fabae*, which employ a set of transporters and permeases during infection that facilitate amino acid uptake (Struck, 2015). *U. maydis* also induces genes coding for amino acid transporters during the early phase of infection and it harbours all enzymes important for amino acid degradation, including those for tryptophan (McCann and Snetselaar, 2008; Lanver *et al*., 2014). Although genes important for amino acid degradation are repressed during appressoria formation (Lanver *et al*., 2014), it is likely that uptake and metabolism of amino acids contributes to growth of *U. maydis* during later stages. In line with those findings, Horst and colleagues (2010) demonstrated that, due to metabolic reprogramming upon *U. maydis* infection, plant‐derived amino acids are relocated to tumor tissue. Therefore, the upregulation of the tryptophan pathway we identified could contribute to feeding during these developmental stages. This might represent a survival strategy of biotrophic pathogens; to rely not only on few nitrogen and carbon sources but to employ a multitude of pathways to obtain nutrients that are abundant in the host and that are of diverse chemical origin, like hexoses, tryptophan, or even SA. Although it was shown that the uptake of sucrose and hexose plays an important role in nutrition during biotrophy (Wahl *et al*., 2010; Schuler *et al*., 2015), the ability to use alternative carbon sources, might ensure that in the absence of the favorable nutrient source the fungus retains its biosynthetic capacity and is able to complete its life cycle.

The transcriptional profiling data of *shy1* in the *rss1* deletion mutant (Fig. 5) indicated that additional factors must be involved in the regulation of Shy1‐dependent SA degradation. Tryptophan and SA degradation might not be entirely co‐regulated and induction might depend on different additional stimuli. This could imply that SA sensing and degradation might have a biological function unrelated to the tryptophan pathway. Therefore, it is tempting to speculate whether *U. maydis* employs SA sensing not only to detect a suitable carbon source *in planta* but also to sense the defense status of the host, to adapt to its environment, and to modulate its own machinery to ensure a successful infection. In this scenario, SA degradation via Shy1 would be an effective mechanism for eliminating SA and resetting Rss1 and other regulators for SA sensing. The lack of an observable virulence phenotype in the *rss1* and *shy1* deletion mutants can be explained by additional or redundant SA sensing and degradation cascades *in planta*. The identification of additional SA sensors, which remained undiscovered in this screen that was based on a single promoter and axenic conditions, and their target genes will provide further insights into why *U. maydis* is able to sense one of the major defense hormones of its host.

Since SA sensing could be a versatile survival strategy of pathogens related to *U. maydis* both as saprophytes as well as in their hosts, we tested whether SA sensing *per se* is conserved among smuts. We showed that *S. reilianum* and *U. hordei* respond to SA with the induction of orthologs of SA‐responsive marker genes (Supporting Information Fig. 9). However, differences in transcriptional responses along with the finding that *U. hordei* likely lost the *rss1* ortholog while retaining the capability to activate *UHOR_shy1*, point towards Rss1‐independent sensing pathways. In line with these findings, we could not identify Rss1‐orthologs by BlastP in SA degrading fungal species including the saprophytes *Trichosporon cutaneum*, the symbiont *Epichloe festucae*, the hemi‐biotroph *Fusarium graminearum* as well as the necrotroph *Sclerotinia sclerotiorum*, suggesting that Rss1‐independent SA‐sensing mechanisms in fungi might exist (Sze and Dagley, 1984; Qi *et al*., 2012; Penn and Daniel, 2013; Ambrose *et al*., 2015).

Several phytohormone nanosensors have been developed enabling the quantification of hormone levels in the cell (Brunoud *et al*., 2012; Jones *et al*., 2014; Waadt *et al*., 2014). Although high‐affinity SA‐binding proteins were identified (dissociation constants of *K*_d_ = 90 nM for SABP2 and *K*_d_ = 45 nM for NPR4) (Du and Klessig, 1997; Fu *et al*., 2012), no SA nanosensor has been established to date. Once the affinity between Rss1 and SA is determined, Rss1 might fill the gap of a missing SA nanosensor and could be employed to assess SA levels inside eukaryotic cells in a quantitative way. The yeast‐based transcriptional activation assay provided already evidence that SA can be detected in a different heterologous eukaryotic system (Fig. 3). Since Rss1 functions as transcriptional activator, quantification of SA could be coupled to a reporter system such as GUS or fluorescence markers. In a further step, it would need to be evaluated whether Rss1 could be used to generate a FRET‐based nanosensor, similar to those established for Auxin and ABA (Brunoud *et al*., 2012; Jones *et al*., 2014; Waadt *et al*., 2014).

With the identification of Rss1 we were not only able to shed light on SA sensing via a multifunctional protein acting as putative receptor and transcriptional activator but also provide the foundation for the generation of valuable tools to assess and monitor cellular SA levels in the future.

## Experimental procedures

### Plasmids, strains and culture conditions

Plasmids were generated according to standard molecular cloning procedures described in Sambrook *et al*. (1989). Primers, plasmids, and cloning strategies employed in this study are compiled in Supporting Information Tables 3 and 4.

*U. maydis* strains used in this study are listed in Supporting Information Table 5. They were generated by gene replacement via homologous recombination with PCR‐generated constructs (Kamper, 2004) or by insertion of p123 derivatives into the *ip* locus (Loubradou *et al*., 2001). Gene deletions and insertions were verified by PCR and/or Southern analysis. To assess SA‐responsiveness of *S. reilianum* and *U. hordei*, the strains SRZ1 (Schirawski *et al*., 2005b) and Uh4875‐4 (Linning *et al*., 2004) were used. For *U. maydis* pathogenicity assays three independent mutants were tested in replicates for virulence on 7‐day‐old maize seedlings of the variety Early Golden Bantam (Olds Seeds, Madison, USA). Disease symptoms were scored 12 days post infection following described protocols (Kamper *et al*., 2006). Statistical analysis was performed using the R statistical environment (R Core Team, 2011)

### UV mutagenesis and cosmid complementation

*U. maydis* SG200P_srg1_mCherry‐3xHA was grown to an exponential phase and adjusted to OD_600nm_ = 1 with H_2_0_dd_. 15 ml of the cell suspension, diluted 1:10^3^ with H_2_0_dd_, were transferred into a petri dish (diameter: 90 mm). To lower surface tension 1 µl 10% Tween 20 was added. UV mutagenesis with a survival rate of 40–50% was achieved by irradiating the cell suspension with 20 mJ using a UV crosslinker (UV Stratalinker 2400; Stratagene, La Jolla, CA, USA). The mutagenized cell suspension was plated on YNB‐N medium supplemented with 2% glucose and 10 mM salicylate. Single colonies were screened for loss of mCherry fluorescence with a widefield stereomicroscope for brightfield and fluorescence (Lumar; Zeiss, Jena, Germany) four days after plating. Images were acquired with the SPOT Pursuit‐XS Monochrome camera controlled by the SPOT Basic Image Capture Software (SPOT Imaging, Sterling Heights, MI, USA). Colonies of cells displaying the expected phenotype were used for inoculation of liquid YEPS_light_ medium (Schirawski *et al*., 2005a) and tested again for loss of fluorescence after spotting in serial dilution on YNB‐N medium (Rabe *et al*., 2013) supplemented with 2% glucose and 10 mM salicylate.

For complementation analysis, SA sensing mutants were transformed with an *U. maydis* cosmid library (Weinzierl, 2001). Colonies were replica plated on YNB‐N plates containing 2% glucose, 10 mM salicylate, and 200 µg ml^−1^ Hygromycin B and screened for rescue of mCherry fluorescence by fluorescence stereomicroscopy. Rescue mutants were grown in YEPS_light_ supplemented with 200 µg ml^−1^ Hygromycin B to retain the autonomously replicating cosmids. Cosmids were re‐isolated by employing the genomic DNA isolation protocol according to Hoffman and Winston (1987) and amplified in *E. coli*. The complementing *U. maydis* fragment, which is inserted in the cosmid, was sequenced with primers 5′ pScos seq and 3′ pScos seq. Sequenced reads were mapped to the *U. maydis* genome by using CLC Main Workbench (Qiagen, Hilden, Germany) to narrow down regions that harbour genes for SA sensing.

### *U. maydis* growth assay on minimal media

Growth assays on salicylate and tryptophan minimal medium were performed as described in Rabe *et al*. (2013). In brief, *U. maydis* strains were grown in YEPS_light_ until OD_600nm_ = 0.8 was reached. Cells were washed three times with H_2_0_dd_ and resuspended in H_2_0_dd_ to OD_600nm_ = 1. Adjusted cultures were spotted in serial dilutions on YNB‐N minimal medium, pH 7.0, supplemented with either 10 mM sodium salicylate or 10 mM L‐tryptophan.

### Yeast‐based assays

AH109 (Clontech/Takara Bio, Saint‐Germain‐en‐Laye, France) was transformed with pGBKT7 or pGADT7 derivatives described in Supporting Information Table 4 using standard protocols (Clontech/Takara Bio, Saint‐Germain‐en‐Laye, France). Strains were grown in selective medium, adjusted to OD_600nm_ = 1 and spotted on indicated media in serial dilutions. Yeast strains used in this study are compiled in Supporting Information Table 6.

To test for transcriptional activation by Rss1, AH109 producing Gal4‐BD‐Rss1 was spotted on selective dropout media plates lacking tryptophan, adenine, and histidine (SD‐Trp‐Ade‐His) and SD‐Trp‐Ade‐His plates supplemented with either 100 µM or 1 mM sodium salicylate, 1 mM L‐tryptophan, or 1 mM anthranilic acid. SD‐Trp was used as growth control plate.

For homodimerization tests of Rss1, in‐frame fusions of Rss1 with Gal4 binding and activation domain, respectively, were produced in yeast. AH109 and Y187 from the Matchmaker™ GAL4 Two‐Hybrid System (Clontech/Takara Bio, Saint‐Germain‐en‐Laye, France) were transformed with either pGADT7 or pGBKT7 derivative containing the respective constructs and mated according to the manufacturer's protocol. Diploids harbouring both plasmids were selected on SD‐Trp‐Leu and homodimerization was assessed by testing growth on high stringency medium (SD‐Trp‐Leu‐Ade‐His).

### Confocal laser scanning microscopy

For colocalization experiments *U. maydis* cells were grown in YNB‐N medium with 10 mM sodium salicylate for 5 h (OD_600nm_ = 0.8). Cells were harvested by centrifugation (2400 g, 5 min) and fixed by addition of 2% formaldehyde in 1x PBS. Samples were incubated for 5 min, centrifuged at 2400 g for 5 min, and washed once with 1x PBS. Afterwards the pellet was resuspended in DAPI (4′,6‐Diamidine‐2′‐phenylindole dihydrochloride) solution (0.5 µg ml^−1^) and incubated for 10 min. After an additional washing step, samples were subjected to confocal microscopy. Colocalization of mCherry‐HA‐Rss1 and DAPI stained nuclei was microscopically assessed by employing an LSM780 Axio Observer confocal laser‐scanning microscope (Zeiss, Jena, Germany) with the following settings: mCherry: Laser DPSS 15 mW, excitation 561 nm, detection 578‐648 nm; DAPI: Laser Diode 25 mW, excitation 405 nm, detection 415–510 nm.

### Quantitative real time PCR and microarray analyses

Quantitative real time PCR was performed as described in Rabe *et al*. (2013). In brief, RNA from axenic culture or from infected plant material was extracted using the TRIzol method according to the manufacturer's protocol (Thermo Fisher Scientific, Waltham, MA, USA), DNA was removed by DNase I treatment (DNA‐*free* Kit; Thermo Fisher Scientific, Waltham, MA, USA) and RNA was reverse transcribed using the RevertAid First Strand cDNA Synthesis Kit (Thermo Fisher Scientific, Waltham, MA, USA). Quantitative real time PCR measurements were performed with a Roche LightCycler® 96 system (Roche Diagnostics, Rotkreuz, Switzerland) according to manufacturer's instructions. Relative expression values were calculated with the 2^−ΔΔCt^ method (Livak and Schmittgen, 2001). Graphical outputs and statistical analyses were performed using GraphPad Prism (v6.0; GraphPad Software, La Jolla, CA, USA).

For global transcriptional profiling, maize plants (Early Golden Bantam) were grown in a plant growth chamber and infected with SG200 or SG200Δrss1 as described previously (Doehlemann *et al*., 2008). Samples were collected in three independently conducted experiments by sampling 12 plants per experiment. To this end, two cm of the third leaf were harvested two cm below the injection point. For RNA isolation, material from the 12 plants was pooled, ground in liquid nitrogen and extracted using TRIzol (Thermo Fisher Scientific, Waltham, MA, USA) according to the manufacturer's protocol. RNA was additionally purified with the RNeasy Kit (Qiagen, Hilden, Germany). Quality and quantity was assessed with the Agilent 2100 Bioanalyzer (Agilent Technologies, Santa Clara, CA, USA). 200 ng purified RNA was subjected to microarray analysis using custom designed Agilent expression microarrays. Chips (8x60K array format) containing *U. maydis* and *Z. mays* gene probes were designed with the online design application eArray (Agilent Technologies, Santa Clara, CA, USA). For probe design the *U. maydis* orfeome (http://www.helmholtz-muenchen.de/en/ibis/institute/groups/fungal-microbial-genomics/resources/mumdb/index.html) and pre‐designed probes from a 4x44K maize gene expression microarray of the eArray AgilentCatalog (Agilent Technologies, Santa Clara, CA, USA) were used. Each Chip included a total of 6,782 sense probes against *U. maydis* genes, along with 6,782 *U. maydis* antisense probes and 42,030 probes against maize genes as well as control probes. The subsequent experimental procedure was performed according to Agilent's Two‐Colour Microarray‐Based Gene Expression Analysis protocol using the Low Input Quick Amp Labelling Kit (Agilent Technologies, Santa Clara, CA, USA). For each individual microarray chip, the respective RNA sample, which was labelled with Cyanine 3‐CTP, was hybridized to the chip together with a common reference pool derived from all samples. The reference pool was labelled with Cyanine 5‐CTP. Data normalization and analysis was conducted in the R statistical environment (R Core Team, 2011) using the limma package (Ritchie *et al*., 2015). Raw expression data for each chip were background normalized by the normexp algorithm. The overall distribution of expression ratios between the green and red channel in each chip was normalized by the loess method. In a subsequent step, the expression data were filtered such that only probes targeting sense transcripts of *U. maydis* were retained for further analysis. This removed noise between samples caused by the plant side and therefore improved the detection of the relatively weak expression changes in the mutant compared to SG200. The log2 expression ratios for the remaining probes were normalized between arrays by the quantile method. A linear model was used to test for significant expression differences between the SG200 and SG200Δrss1 samples. Since location‐dependent effects on plant growth between the three replicates could be observed in the plant growth chamber, the estimation of a “sampling date” effect was included in the model to subtract background noise between replicates. Differential expression was determined by the limma ebayes function. *P*‐values were corrected for multiple testing by the Benjamini‐Hochberg method (Benjamini and Hochberg, 1995). Expression data were submitted to GeneExpressionOmnibus (http://www.ncbi.nlm.nih.gov/geo/) under the accession number GSE83576.

### Next generation illumina sequencing

To sequence genomes of mutant strains, their genomic DNA was subjected to Next Generation Illumina Sequencing. To this end, genomic DNA was extracted according to the method of Hoffman and Winston (1987) and purified by an additional purification step using the MasterPure Complete DNA and RNA Purification Kit (Epicentre, Madison, WI, USA). DNA concentration was determined by PicoGreen measurements (Thermo Fisher Scientific, Waltham, MA, USA). 1 µg genomic DNA was subsequently sheared in micro AFA tubes using an S220 focused ultra‐sonicator with AFA technology (Covaris, Woburn, MA, USA). Average fragmentation size was assessed by performing a Fragment Analyzer run with the High Sensitivity NGS Fragment Analysis Kit (Advanced Analytical Technologies, Ankeny, IA, USA). Fragmented DNA was subsequently converted into indexed libraries for Next Generation Sequencing using the NEBNext®Ultra™ DNA Library Prep Kit for Illumina®. The library was prepared according to the manufacturer's protocol. Quality control and Illumina 125 bp paired end sequencing on a HiSeq 2500 instrument was carried out by the Next Generation Sequencing Facility (VBCF, Vienna, Austria).

Removal of adapter contamination and trimming of low quality 3′ read ends was performed with BBDUK (BBMap ‐ Bushnell B. ‐ sourceforge.net/projects/bbmap/) and Trimmomatic (v0.33; Bolger *et al*., 2014), respectively. Paired‐end reads were mapped to the *U. maydis* reference genome with BWA (v0.7.8; Li and Durbin, 2009), while duplicated reads were removed with Picard (v1.101; http://broadinstitute.github.io/picard). Local realignment around indels and base recalibration were done with GATK (v3.5; DePristo *et al*., 2011). All format conversions were done with Samtools (v0.1.18; Li *et al*., 2009). Joint genotyping of all sequenced strains was performed with GATK/UnifiedGenotyper in SNP mode setting the ploidy parameter to 1 for haploid individuals. Variants common to all strains, including the progenitor SG200P_srg1_mCherry‐3xHA, were discarded for considering that those mutations with respect to the reference existed before UV mutagenesis. In addition, variant positions with quality ≤ 50 or supported by more than one read with mapping quality zero were filtered out in all strains. At the sample level, variants where the log2 of the ratio between the read depth and the median coverage of the strain was either > 0.5 or < −0.5 were excluded from the analysis. Mapped reads were visualized with IGV (v2.3.57; Robinson *et al*., 2011; Thorvaldsdottir *et al*., 2013). Sequencing data were submitted to NCBI under the BioProject accession number PRJNA326324, Study ID SRP076835.

### Bioinformatic analyses

Gene and protein sequences of *U. maydis*, *S. reilianum*, and *U. hordei* as well as gene and protein information were obtained from the MIPS *Ustilago maydis* database (http://www.helmholtz-muenchen.de/en/ibis/institute/groups/fungal-microbial-genomics/resources/mumdb/index.html), the MIPS *Sporisorium reilianum* database (http://www.helmholtz-muenchen.de/ibis/institute/groups/fungal-microbial-genomics/resources/msrdb/index.html), and the MIPS *Ustilago hordei* database (http://www.helmholtz-muenchen.de/ibis/institute/groups/fungal-microbial-genomics/resources/muhdb/index.html). The Rss1 protein sequences of *S. scitamineum* (GeneBank Accession No. CDU25879) and *M. pennsylvanicum* (GeneBank Accession No. CDI53350) were taken from the ‘National Center of Biotechnology Information’ (NCBI; www.ncbi.nlm.nih.gov/). BlastP (Basic Local Alignment Search Tool) version 2.225 was employed for the identification of potential Rss1 orthologs using standard search parameters (Altschul *et al*., 1990). Homologous amino acid sequences were compared with CLC Main Workbench (v7.0.2; Qiagen, Hilden, Germany) using progressive alignment algorithms. Protein domains were identified with the Simple Modular Architecture Research Tool ‚SMART’ (Schultz *et al*., 1998; Letunic *et al*., 2015) and nuclear localizations were predicted with NucPred (Brameier *et al*., 2007). PEST motifs were determined with epestfind (v5.0.0; Rice *et al*., 2000)

## Authors contributions

Conceived and designed the experiments: FR and AD. Performed the wet bench experiments: FR, DS, LB, and FN. Performed bioinformatics analyses: ACE and FAR. Wrote the manuscript: FR and AD with input from all co‐authors. Directed the project: AD.

## Authors declaration

The Authors declare that there is no conflict of interest in the research.

## Supporting information

Supporting Information
