# Supplementary material for: Phytohormone sensing in the biotrophic fungus Ustilago maydis – the dual role of the transcription factor Rss1
Source: Mol Microbiol. 2016 Aug 8;102(2):290–305. doi: 10.1111/mmi.13460 (PMC5082525; doi:10.1111/mmi.13460)
Supplement: Supplementary file 1 — Supporting Information [file MMI-102-290-s001.pdf]

Supporting information file

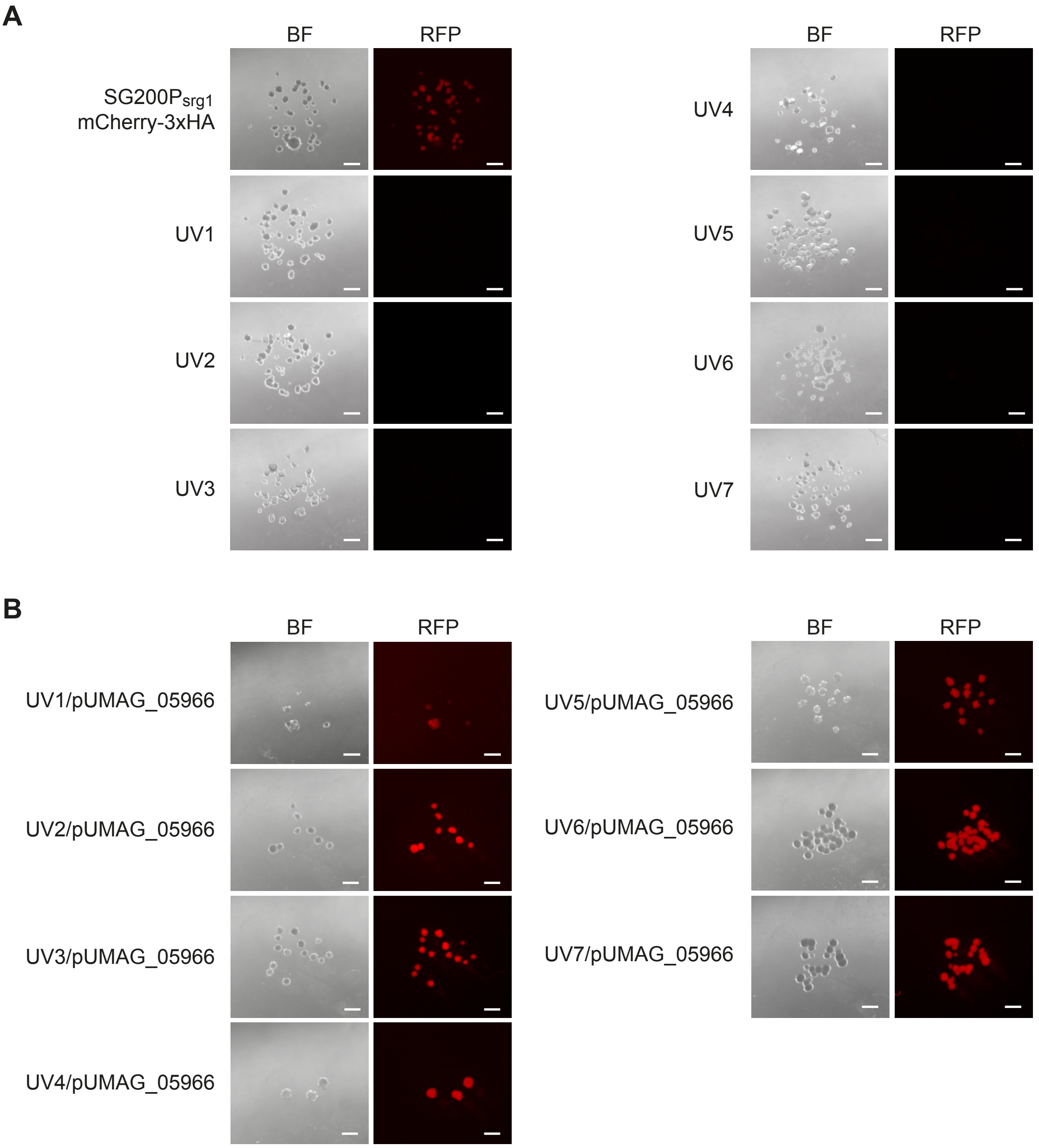

Suppl. Fig. 1: UV mutants do not show mCherry fluorescence and pUMAG_05966 restores fluorescence of these mutant strains. UV mutants, before **(A)** and after **(B)** transformation with pUMAG_05966, were spotted on YNB-N medium supplemented with glucose and 10 mM sodium salicylate (YNB-N Glc + SA). In case of the strains harboring pUMAG_05966 (B), 200 µg ml^{-1} Hygromycin B was added to the medium to ensure that transformants retained the autonomously replicating pUMAG_05966 plasmid. As positive control, SG200P_{srg1}mCherry-3xHA was included in the analysis and spotted on YNB-N Glc + SA. Fluorescence of colonies was assessed by fluorescence stereomicroscopy four days after spotting (BF = brightfield channel; RFP = RFP channel). Scale bars: 1 mm. While for UV mutants, no mCherry signal could be detected, strains transformed with pUMAG_05966 showed restored fluorescence.

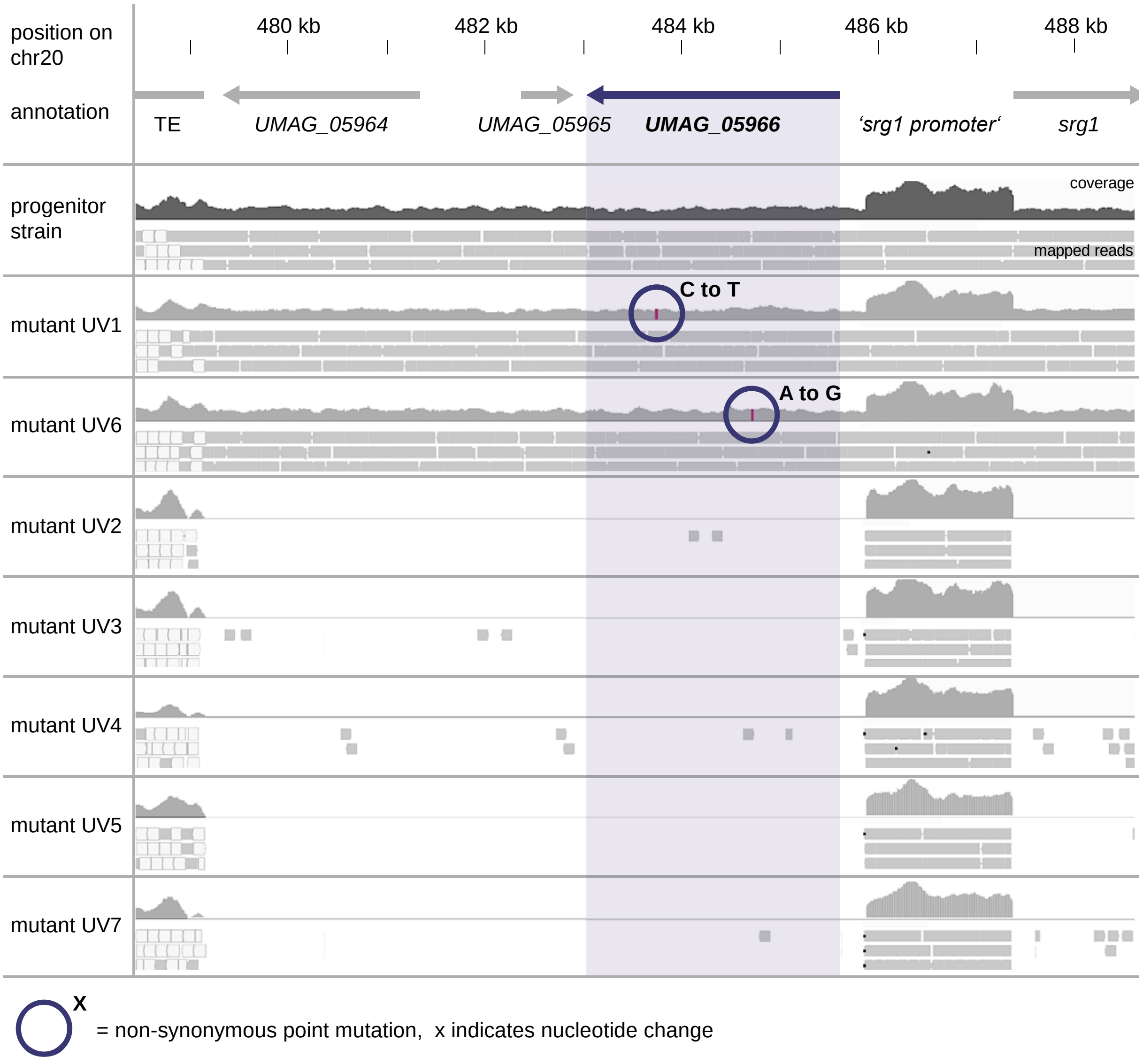

Suppl. Fig. 2: Verification of UV mutants by Next Generation Illumina Sequencing. Genomes of UV mutants with loss of fluorescence and the progenitor strain SG200P$_{srg1}$mCherry-3xHA were sequenced by Illumina HiSeq 2500 125 bp paired-end sequencing and reads were mapped against the genome of the reference strain 521 (Kamper et al., 2006). For each mutant, the upper track represents coverage, while the lower track displays a fraction of mapped reads. Depicted is the genomic region of chromosome 20 including *UMAG_05966*. A transposable element was identified in this region and labeled TE. Grey blocks represent aligned sequence reads, white blocks represent reads that mapped ambiguously. Mutant UV1 and UV6 each harbor a single non-synonymous mutation in *UMAG_05966*, marked with a blue circle. The resulting nucleotide change is indicated above each circle. The point mutation in UV1 caused an amino acid change of glycine to aspartic acid at position 629 (G$_{629}$D). The mutation in UV6 resulted in a change of phenylalanine to serine at amino acid position 303 (F$_{303}$S). All other mutants lack the entire end of chromosome 20 including *UMAG_05966.* High coverage for 1500 bp *srg1* promoter is caused by reads originating from P$_{srg1}$mCherry-3xHA, which was inserted in multiple copies into the *ip* locus on chromosome 2.

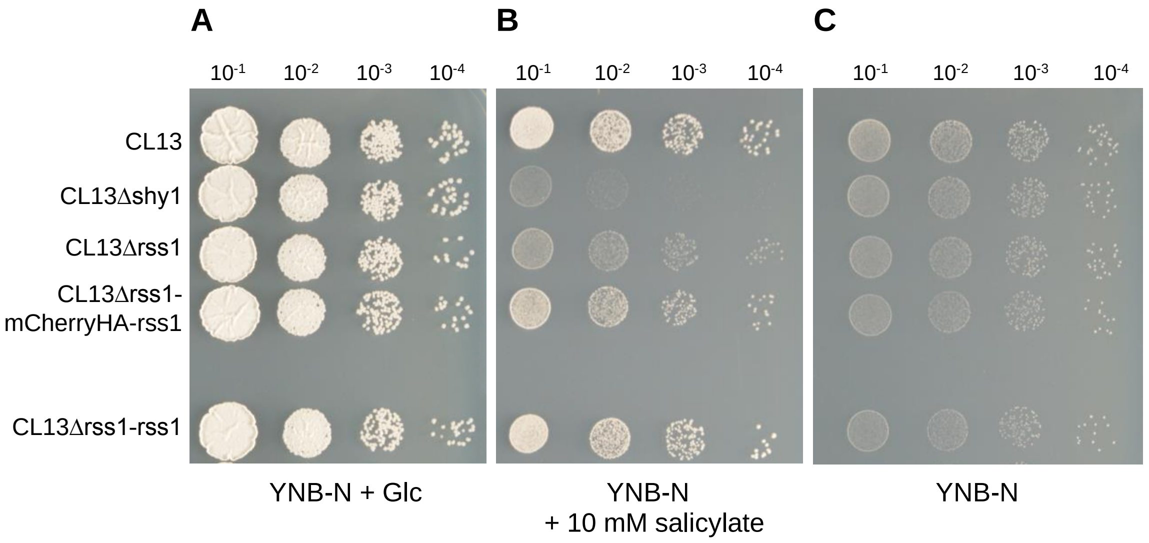

Suppl. Fig. 3: The *UMAG_05966* deletion mutant shows Δ*shy1*-like growth attenuation on medium with salicylate as sole carbon source. Growth of CL13, CL13Δshy1, CL13Δrss1, CL13Δrss1-mCherryHA-rss1 and CL13Δrss1-rss1 on YNB-N supplemented with 2% glucose (Glc) **(A)**, on YNB-N with 10 mM sodium salicylate **(B)**, and without any carbon source **(C)**. Growth of a *UMAG_05966* deletion mutant (CL13Δrss1) was attenuated similar to CL13Δshy1 on salicylate minimal medium. The growth defect was partially rescued by ectopic integration of P_{UMAG_05966}*mCherryHA-UMAG_05966* (CL13Δrss1-mCherryHA-rss1). Full rescue of the mutant phenotype was obtained after an ectopic integration of untagged *UMAG_05966* under control of the *UMAG_05966* promoter (CL13Δrss1-rss1).

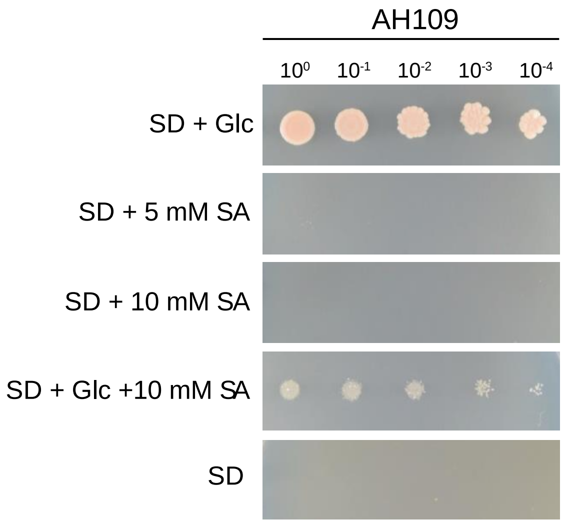

Suppl. Fig. 4: AH109 is not capable of using salicylate as sole carbon source and it is severely growth-attenuated in the presence of SA. AH109 was spotted in serial dilutions on indicated media. No growth was detected on SD medium containing 5 or 10 mM salicylate as sole carbon source (SD + 5 mM SA; SD + 10 mM SA). Addition of 10 mM salicylate to glucose-containing SD medium (SD + Glc + 10 mM SA) resulted in severe growth retardation.

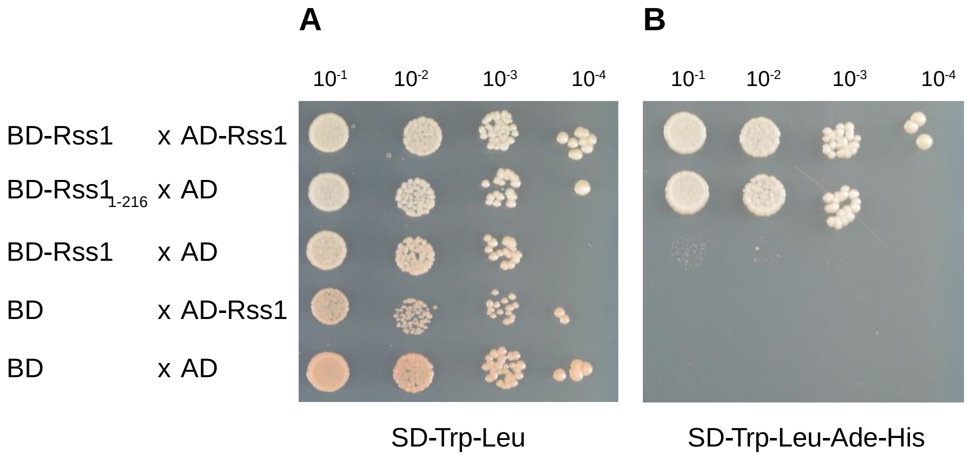

Suppl. Fig. 5: Rss1 forms a homodimer. Yeast strains producing Rss1 fused to the binding (BD) and activation domain (AD) of the Gal4 transcription factor, respectively, as well as controls with the autoactive version of Rss1, $Rss1_{1\text{-}216}$, and without any Rss1 fusion were spotted in serial dilutions on SD-Trp-Leu **(A)** to select for cells containing both plasmids and on SD-Trp-Leu-Ade-His **(B)** to assay for Rss1 dimerization.

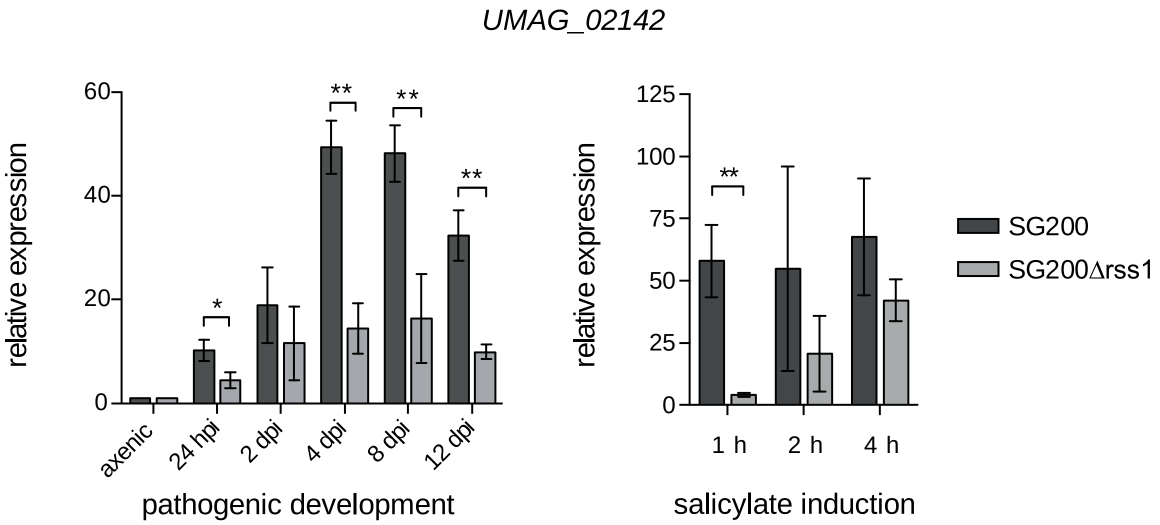

Suppl. Fig. 6: *UMAG_02142* is transcriptionally induced upon SA treatment and during pathogenic development and its levels are significantly reduced in the absence of *rss1*. Transcript levels of *UMAG_02142* were quantified in SG200 and SG200Δrss1 by real time PCR. RNA was isolated from the indicated life cycle stages of pathogenic development ('pathogenic development', left panel) and from a time course after shift to YNB-N medium containing 2% glucose and 10 mM salicylate ('salicylate induction', right panel). Constitutively expressed *peptidyl-prolyl isomerase* (*ppi*) was used for normalization. Transcript levels were either compared to levels in axenic culture grown in $YEPS_{light}$ medium (left panel) or grown in YNB-N medium with 2% glucose (right panel). Expression levels in axenic culture (left panel) or glucose-grown cells (right panel) were set to 1.0. Error bars depict the standard deviation calculated from three independent biological replicates (n=3). Significance was calculated with unpaired *t* test comparing transcript levels of indicated genes in SG200 with those in SG200Δrss1, * $p \leq 0.05$, ** $p \leq 0.01$. For transcriptional profiling of different life cycle stages, RNA extracted from twelve infected plants per time point and replicate was used.

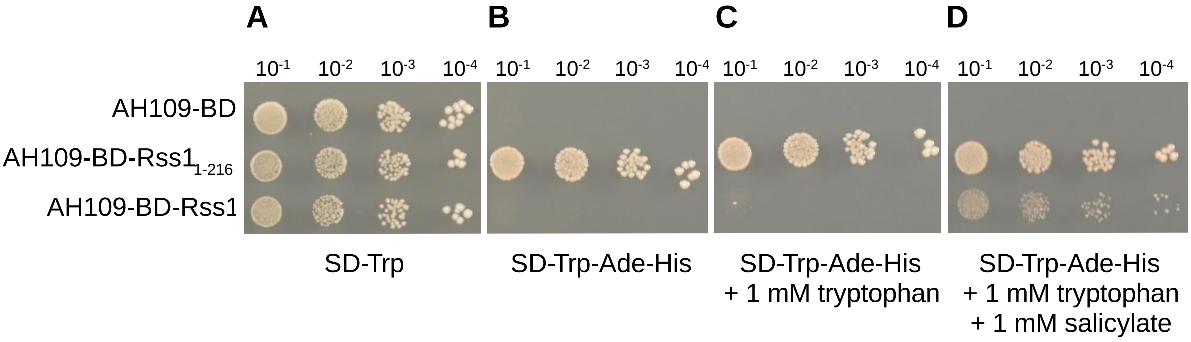

Suppl. Fig. 7: Tryptophan cannot activate Rss1 in yeast. AH109 expressing *Gal4-BD* (AH109-BD, negative control), *Gal4-BD-rss1*$_{1\text{-}216}$ (AH109-BD-Rss1$_{1\text{-}216}$, positive control) and *Gal4-BD-rss1* (AH109-BD-Rss1), respectively, were spotted in serial dilutions on SD-Trp (growth control) **(A)**, on SD-Trp-Ade-His **(B)**, SD-Trp-Ade-His with 1 mM tryptophan **(C)**, and SD-Trp-Ade-His with 1 mM tryptophan and 1 mM sodium salicylate **(D)**. Growth of AH109-BD-Rss1 on high stringency medium was only visible in the presence of salicylate and not with tryptophan alone.

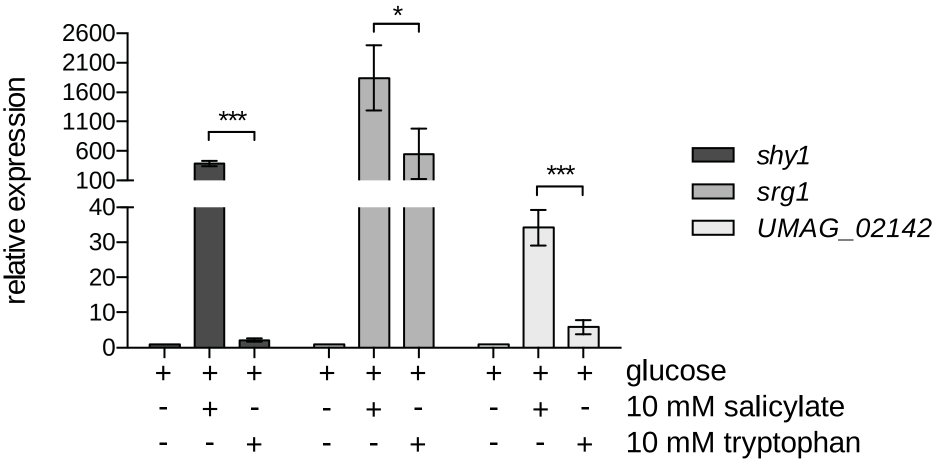

Suppl. Fig. 8: *shy1*, *srg1*, and *UMAG_02142* are transcriptionally induced in the presence of tryptophan but significantly less than compared to SA. Transcript levels of *shy1*, *srg1*, and *UMAG_02142* were determined by quantitative real time PCR one hour after shift to YNB-N supplemented with 2% glucose and either 10 mM salicylate or 10 mM tryptophan. Levels were compared to those of cells grown in YNB-N supplemented with 2% glucose. Levels in glucose-treated control cells are set to 1.0. Constitutively expressed *peptidyl-prolyl isomerase* (*ppi*) was used for normalization. Significance was calculated with unpaired *t* test comparing expression values with those of glucose-treated control cells, * $p \leq 0.05$, *** $p \leq 0.001$. Transcript levels of the tested SA-responsive genes were induced after shift to tryptophan-containing medium but expression is significantly less than after SA treatment ($p < 0.033$).

A

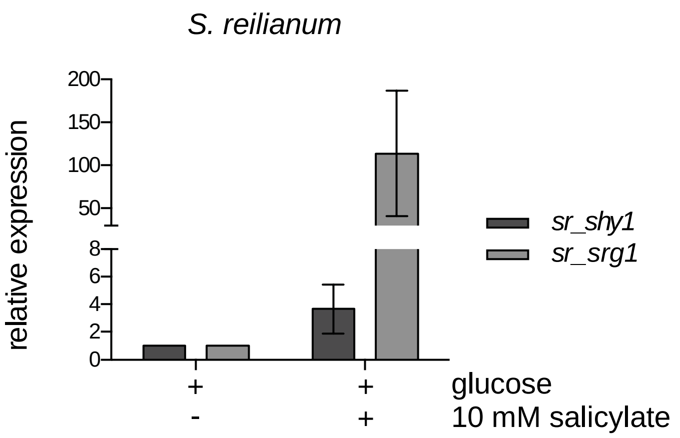

B

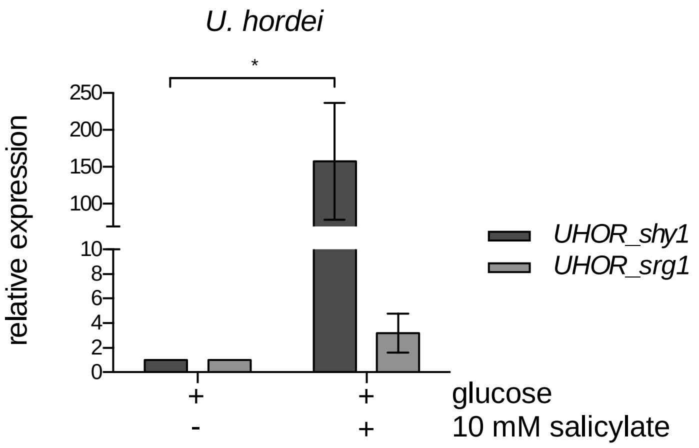

Suppl. Fig. 9: SA sensing is conserved among smuts. *S. reilianum* SRZ1 and *U. hordei* Uh4875-4 were grown in $YEPS_{light}$ liquid medium, shifted to YNB-N supplemented with 2% glucose or 2% glucose and 10 mM salicylate, and harvested after 1 hour. Transcript levels of *sr_shy1 (sr13139)* and *sr_srg1* (*sr16595*) from *S. reilianum* **(A)** and *UHOR_shy1* (*UHOR_03215)* and *UHOR_srg1* (*UHOR_08490*) from *U. hordei* **(B)** were determined by quantitative real time PCR after RNA isolation and cDNA synthesis. Error bars depict standard deviation (n=3). Constitutively expressed *peptidyl-prolyl isomerase (ppi)* genes *sr1196* and *UHOR_05685* were used for normalization. Transcript levels were compared to those of cells grown in YNB-N with glucose. Expression levels of glucose-treated control cells were set to 1.0. Significance was calculated with unpaired *t* test comparing expression values with those of glucose-treated control cells, * $p \leq 0.05$.

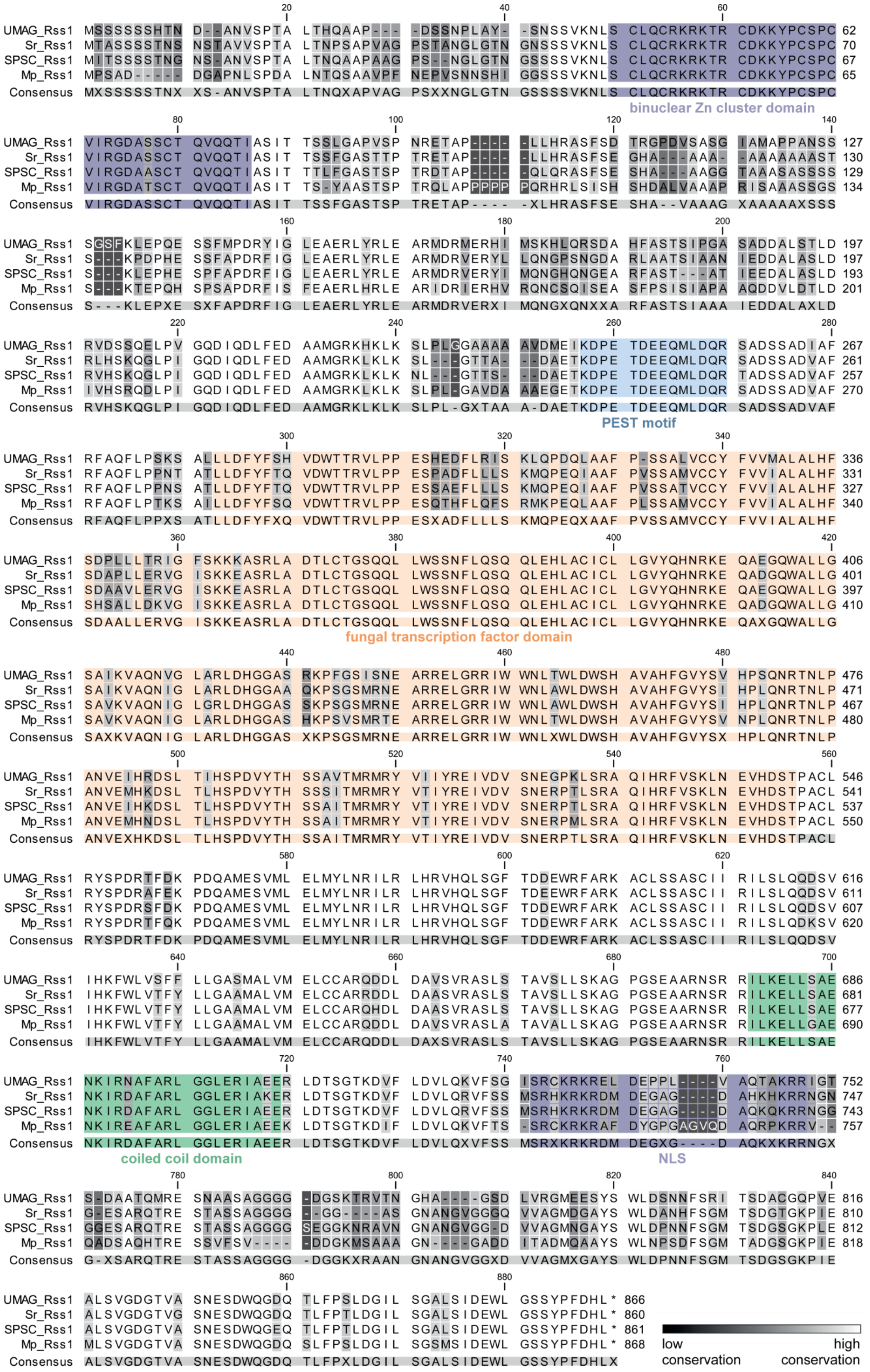

20 40 60
UMAG_Rss1 MSSSSSSHTN D--ANVSPTA LTHQAAP--- -DSSNPLAY- -SNSSVKNLS CLQCRKRKTR CDKKYPCSPC 62
Sr_Rss1 MTASSSSTNS NSTAVVSPTA LTNPSAPVAG PSTANGLGTN GNSSSVKNLS CLQCRKRKTR CDKKYPCSPC 70
SPSC_Rss1 MITSSSSTNG NS-ANVSPTA LTNQAAPAAG PS--NGLGTN GSSSSVKNLS CLQCRKRKTR CDKKYPCSPC 67
Mp_Rss1 MPSAD----- DGAPNLSPDA LNTQSAAVPF NEPVSNNSHI GGSSSVKNLS CLQCRKRKTR CDKKYPCSPC 65
Consensus MXSSSSSTNX XS-ANVSPTA LTNQXAPVAG PSXXNGLGTN GSSSSVKNLS CLQCRKRKTR CDKKYPCSPC
binuclear Zn cluster domain
80 100 120 140
UMAG_Rss1 VIRGDASSCT QVQQTIASIT TSSLGAPVSP NRETAP---- -LLHRASFSD TRGPDVSASG IAMAPPANSS 127
Sr_Rss1 VIRGDASSCT QVQQTIASIT TSSFGASTTP TRETAP---- -LLHRASFSE GHA---AAA- -AAAAAAAST 130
SPSC_Rss1 VIRGDAASCT QVQQTIASIT TTLFGASTSP TRDTAP---- -QLQRASFSE SHA---AAGG TAAASASSSS 129
Mp_Rss1 VIRGDATSCT QVQQTIASIT TS-YAASTSP TRQLAPPPPP PQRHRLSISH SHDALVAAAP RISAAASSGS 134
Consensus VIRGDASSCT QVQQTIASIT TSSFGASTSP TRETAP---- -XLHRASFSE SHA--VAAAG XAAAAAXSSS
160 180 200
UMAG_Rss1 SGSFKLEPQE SSFMPDRYIG LEAERLYRLE ARMDRMERHI MSKHLQRSDA HFASTSIPGA SADDALSTLD 197
Sr_Rss1 S---KPDPHE SSFAPDRFIG LEAERLYRLE ARMDRVERYL LQNGPSNGDA RLAATSIAAN IEDDALASLD 197
SPSC_Rss1 S---KLEPHE SPFAPDRFIG LEAERLYRLE ARMDRVERYI MQNGHQNGEA RFAST---AT IEEDALASLD 193
Mp_Rss1 S---KTEPQH SPSAPDRFIS FEAERLHRLE ARIDRIERHV RQNCSQISEA SFPSISIAPA AQDDVLDTLD 201
Consensus S---KLEPXE SXFAPDRFIG LEAERLYRLE ARMDRVERXI MQNGXQNXXA RFASTSIAAA IEDDALAXLD
220 240 260 280
UMAG_Rss1 RVDSSQELPV GQDIQDLFED AAMGRKHKLK SLPLGGAAAA AVDMEIKDPE TDEEQMLDQR SADSSADIAF 267
Sr_Rss1 RLHSKQGLPI GQDIQDLFED AAMGRKLKLK SL---GTTA- --DAETKDPE TDEEQMLDQR SADSSADVAF 261
SPSC_Rss1 RVHSKQGLPI GQDIQDLFED AAMGRKLKLK NL---GTTS- --DAETKDPE TDEEQMLDQR TADSSADVAF 257
Mp_Rss1 IVHSRQDLPI GQDIQDLFED AAMGRKLKLK SLPL-GAVDA AAEGETKDPE TDEEQMLDQR SADSSADVAF 270
Consensus RVHSKQGLPI GQDIQDLFED AAMGRKLKLK SLPL-GXTAA A-DAETKDPE TDEEQMLDQR SADSSADVAF
PEST motif
300 320 340
UMAG_Rss1 RFAQFLPSKS ALLLDFYFSH VDWTTRVLPP ESHEDFLRIS KLQPDQLAAF P-SSALVCCY FVVMALALHF 336
Sr_Rss1 RFAQFLPPNT ATLLDFYFTQ VDWTTRVLPP ESPADFLLLS KMQPEQIAAF PVSSAMVCCY FVVIALALHF 331
SPSC_Rss1 RFAQFLPPNS ATLLDFYFTQ VDWTTRVLPP ESSAEFLLLS KMQPEQIAAF PVSSATVCCY FVVIALALHF 327
Mp_Rss1 RFAQFLPTKS AILLDFYFSQ VDWTTRVLPP ESQTHFLQFS RMKPEQLAAF PLSSAMVCCY FVVIALALHF 340
Consensus RFAQFLPPXS ATLLDFYFXQ VDWTTRVLPP ESXADFLLLS KMQPEQXAAF PVSSAMVCCY FVVIALALHF
360 380 400 420
UMAG_Rss1 SDPLLLTRIG FSKKKASRLA DTLCTGSQQL LWSSNFLQSQ QLEHLACICL LGVYQHNRKE QAEGQWALLG 406
Sr_Rss1 SDAPLLERVG ISKKEASRLA DTLCTGSQQL LWSSNFLQSQ QLEHLACICL LGVYQHNRKE QADGQWALLG 401
SPSC_Rss1 SDAAVLERVG ISKKEASRLA DTLCTGSQQL LWSSNFLQSQ QLEHLACICL LGVYQHNRKE QAEGQWALLG 397
Mp_Rss1 SHSALLDKVG ISKKEASRLA DTLCTGSQQL LWSSNFLQSQ QLEHLACICL LGVYQHNRKE QADGQWALLG 410
Consensus SDAALLERVG ISKKEASRLA DTLCTGSQQL LWSSNFLQSQ QLEHLACICL LGVYQHNRKE QAXGQWALLG
fungal transcription factor domain
440 460 480
UMAG_Rss1 SAIKVAQNVG LARLDHGGAS RKPFGSISNE ARRELGRRIW WNLTWLDWSH AVAHFGVYSV HPSQNRTNLP 476
Sr_Rss1 SAIKVAQNIG LARLDHGGAA QKPSGSMRNE ARRELGRRIW WNLAWLDWSH AVAHFGVYSI HPLQNRTNLP 471
SPSC_Rss1 SAVKVAQNIG LGRLDHGGAS SKPSGSMRNE ARRELGRRIW WNLAWLDWSH AVAHFGVYSI HPLQNRTNLP 467
Mp_Rss1 SAVKVAQNIG LARLDHGGAS HKPSVSMRTE ARRELGRRIW WNLTWLDWSH AVAHFGVYSV NPLQNRTNLP 480
Consensus SAXKVAQNIG LARLDHGGAS XKPSGSMRNE ARRELGRRIW WNLXWLDWSH AVAHFGVYSX HPLQNRTNLP
500 520 540 560
UMAG_Rss1 ANVEIHRDSL TIHSPDVYTH SSAVTMRMRY VIIYREIVDV SNEGPKLSRA QIHRFVSKLN EVHDSTPACL 546
Sr_Rss1 ANVEMHKDSL TLHSPDVYTH SSSITMRMRY VTIYREIVDV SNERPTLSRA QIHRFVSKLN EVHDSTPACL 541
SPSC_Rss1 ANVEIHKDSL TLHSPDVYTH SSAITMRMRY VTIYREIVDV SNERPTLSRA QIHRFVSKLN EVHDSTPACL 537
Mp_Rss1 ANVEMHNDSL TLHSPDVYTH SSAITMRMRY VTIYREIVDV SNERPMLSRA QIHRFVSKLN EVHDSTPACL 550
Consensus ANVEXHKDSL TLHSPDVYTH SSAITMRMRY VTIYREIVDV SNERPTLSRA QIHRFVSKLN EVHDSTPACL
580 600 620
UMAG_Rss1 RYSPDRTFDK PDQAMESVML ELMYLNRILR LHRVHQLSGF TDDEWRFARK ACLSSASCII RILSLQQDSV 616
Sr_Rss1 RYSPDRAFEK PDQAMESVML ELMYLNRILR LHRVHQLSGF TDEEWRFARK ACLSSASCII RILSLQQDSV 611
SPSC_Rss1 RYSPDRSFDK PDQAMESVML ELMYLNRILR LHRVHQLSGF TDDEWRFARK ACLSSASCII RILSLQQDSV 607
Mp_Rss1 RYSPDRTFQK PDQAMESVML ELMYLNRILR LHRVHQLSGF TDDEWRFARK ACLSSASCII RILSLQDKSV 620
Consensus RYSPDRTFDK PDQAMESVML ELMYLNRILR LHRVHQLSGF TDDEWRFARK ACLSSASCII RILSLQQDSV
640 660 680 700
UMAG_Rss1 IHKFWLVSFF LLGASMALVM ELCCARQDDL DAVSVRASLS TAVSLLSKAG PGSEAARNSR RILKELLSAE 686
Sr_Rss1 IHKFWLVTFY LLGAAMALVM ELCCARRDDL DAASVRASLS TAVSLLSKAG PGSEAARNSR RILKELLSAE 681
SPSC_Rss1 IHKFWLVTFY LLGAAMALVM ELCCARQHDL DAASVRASLS TAVSLLSKAG PGSEAARNSR RILKELLSAE 677
Mp_Rss1 IHKFWLVTFY LLGAAMALVM ELCCARQDDL DAVSVRASLA TAVALLSKAG PGSEAARNSR RILKELLGAE 690
Consensus IHKFWLVTFY LLGAAMALVM ELCCARQDDL DAXSVRASLS TAVSLLSKAG PGSEAARNSR RILKELLSAE
720 740 760
UMAG_Rss1 NKIRNAFARL GGLERIAEER LDTSGTKDVF LDVLQKVFSG ISRCKRKREL DEPPL----V AQTAKRRIGT 752
Sr_Rss1 NKIRDAFARL GGLERIAKER LDTSGTKDVF LDVLQRVFSS MSRHKRKRDM DEGAG----D AHKHKRRNGN 747
SPSC_Rss1 NKIRDAFARL GGLERIAEER LDTSGTKDVF LDVLQRVFSS MSRHKRKRDM DEGAG----D AQKQKRRNGG 743
Mp_Rss1 NKIREAFARL GGLERIAEEK LDTSGTKDIF LDVLQKVFTS -SRCKRKRAF DYGPGAGVQD AQRPKRRV-- 757
Consensus NKIRDAFARL GGLERIAEER LDTSGTKDVF LDVLQXVFSS MSRXKRKRDM DEGXG----D AQKXKRRNGX
coiled coil domain
NLS
780 800 820 840
UMAG_Rss1 S-DAATQMRE SNAASAGGGG -DGSKTRVTN GHA----GSD LVRGMEESYS WLDSNNFSRI TSDACGQPVE 816
Sr_Rss1 G-ESARQTRE STASSAGGGG --GG----AS GNANGVGGGQ VVAGMDGAYS WLDANHFSGM TSDGTGKPIE 810
SPSC_Rss1 GGESARQTRE STASSAGGGG SEGGKNRAVN GNANGVGG-D VVAGMNGAYS WLDPNNFSGM TSDGSGKPLE 812
Mp_Rss1 QADSAQHTRE SSVFSV---- -DDGKMSAAA GN----GADD ITADMQAAYS WLNPSDFSGM TADGSGKPIE 818
Consensus G-XSARQTRE STASSAGGGG -DGGKXRAAN GNANGVGGXD VVAGMXGAYS WLDPNNFSGM TSDGSGKPIE
860 880
UMAG_Rss1 ALSVGDGTVA SNESDWQGDQ TLFPSLDGIL SGALSIDEWL GSSYPFDHL* 866
Sr_Rss1 ALSVGDGTVG SNESDWQGDQ TLFPTLDGIL SGALSIDEWL GSSYPFDHL* 860
SPSC_Rss1 ALSVGDGTVA SNESDWQGEQ TLFPTLDGIL SGALSIDEWL GSSYPFDHL* 861
Mp_Rss1 MLSVGDGTVA SNESDWQGDQ SLFPSLDGIL SGSMSIDEWL GSSYPFDHL* 868
Consensus ALSVGDGTVA SNESDWQGDQ TLFPXLDGIL SGALSIDEWL GSSYPFDHLX
low conservation
high conservation

Suppl. Fig. 10: Rss1 is conserved between *U. maydis*, *S. reilianum, S. scitamineum*, and *M. pennsylvanicum*. Rss1 orthologs show an overall conservation with the highly conserved Zn(II)$_2$Cys$_6$-DNA binding domain (purple), putative fungal transcription factor domain (orange), PEST motif (blue), coiled coil domain (green), and NLS (purple). Only N- and C-termini are less conserved. For simplification *U. maydis* Rss1 is named UMAG_Rss1, Sr16594 from *S. reilianum* Sr_Rss1, SPSC_06050 from *S. scitamineum* SPSC_Rss1, and Bn877_02897 from *M. pennsylvanicum* Mp_Rss1. Grey scale depicts conservation of residues (transparent background = highly conserved residues, black background = non-conserved residues). X stands for ambiguous amino acids.

Supplemental Table 1: Overview of mutations and large chromosomal deletions/alterations found in UV mutants.

mutant	individual SNPs	SNP-containing genes shared between mutants[#]	non-synonymous mutation in *UMAG_05966*?	partial loss of chr20, including *UMAG_05966*?	Strain with aneuploidy?*
**UV1**	5	no	yes, C to T, leading to $G_{629}D$	no	no
**UV2**	26	*UMAG_11191*, silent mutation	no	yes	yes; chr20
**UV3**	4	no	no	yes	yes; chr13 partial, chr19
**UV4**	4	no	no	yes	yes; chr21 partial, chr22
**UV5**	9	*UMAG_11191*, W to Stop	no	yes	yes; chr12 partial
**UV6**	21	no	yes, A to G, leading to $F_{303}S$	no	no
**UV7**	9	no	no	yes	yes; chr19 partial

[#] Genomes were scanned for SNP-containing regions that are shared between mutants. The analysis did not reveal any SNP-containing promoter that is shared between mutants. Shared SNP-containing genes are listed for each mutant, and the type of mutation is indicated below.

* Chromosomal region with twice the average coverage indicates duplication (aneuploidy). Chromosomes with increased coverage are listed.

Supplemental Table 2: Constructs and conditions tested for Rss1 production.[1]

Construct	Expression system	Purification method[2]	Culture condition	Comments
**Strep-Rss1$_{codonopt.}$**	*E.coli* BL21	Strep-Tactin purification	100 µM $ZnCl_2$, +/- 5 mM SA[3]	*E.coli* codon-optimized version of Rss1; no full-length protein could be purified
**StrepHA-GFP-PP-Rss1$_{codonopt.}$**	*E.coli* BL21	Strep-Tactin purification	100 µM $ZnCl_2$[4]	*E.coli* codon-optimized version of Rss1; no full-length protein could be purified
**6xHis-MBP-PP-Strep-GFP-Rss1$_{codonopt.}$**	*E.coli* BL21, *E.coli* Rosetta 2	Ni-NTA affinity purification	100 µM $ZnCl_2$[4]	Full length protein could be purified but aggregated after cleavage of 6xHis-MBP tag.

Construct	Expression system	Purification method[2]	Culture condition	Comments
**StrepHA-GFP-PP-Rss1**	*U. maydis* SG200	Strep-Tactin purification, +/- 1 mM SA, +/- proteasome inhibitor MG132 to prevent potential degradation	Harvest of culture with OD_{600} of 1; expression was pre-checked by nuclear GFP fluorescence	Construct is under control of the strong constitutively active *oma* promoter (Flor-Parra et al. 2006). Overexpression led to speckle-like structures in the nucleus and insoluble protein indicating protein aggregation.

[1] Only N-terminal fusions were included in this table since the addition of C-terminal tags leads to biological inactive Rss1 versions that fail to rescue the observed growth defect on SA minimal medium (data not shown).

[2] Since Rss1 represents a potential zinc cluster protein, EDTA was omitted and 100 µM $ZnCl_2$ was added for purification.

[3] Induction conditions: 1 mM IPTG added to culture in exponential phase (OD_{600} = 0.5 - 0.7), 7 h / overnight (18 h), 16°C / 20°C

[4] Induction condition: 1 mM IPTG added to culture in exponential phase (OD_{600} = 0.5 - 0.7), overnight, 20°C

Supplemental Table 3: Primers used in this study.*

Name	Sequence (5'-3')	Restriction site	Application
5' RT ppi	ACATCGTCAAGGCTATCG		real time PCR primer for *ppi* (*UMAG_03726*)
3' RT ppi	AAAGAACACCGGACTTGG		real time PCR primer for *ppi* (*UMAG_03726*)
5' RT shy1	TGGCGACACGTATGAGTTCC		real time PCR primer for *UMAG_05230*
3' RT shy1	CTCGGCGTCCAAATCGTAAT		real time PCR primer for *UMAG_05230*
5' RT srg1	CAAGAAGCAGACGTGCATCC		real time PCR primer for *UMAG_05967*
3' RT srg1	CAGCCTCGATGTTGCTGGTA		real time PCR primer for *UMAG_05967*
5' RT sr_ppi	GTCCTTCCACCGTGTCATCC		real time PCR primer for *ppi* (*sr1196*) from *S. reilianum*
3' RT sr_ppi	GTTCTCGTCGGCAAACTTGG		real time PCR primer for *ppi* (*sr1196*) from *S. reilianum*
5' RT sr_shy1	ACTTTGCCTCGTACAGCTCC		real time PCR primer for *sr_shy1* (*sr13139)* from *S. reilianum*
3' RT sr_shy1	GATGTACGAGGTGAGCGAGG		real time PCR primer for *sr_shy1* (*sr13139)* from *S. reilianum*

**Name**	**Sequence (5'-3')**	**Restriction site**	**Application**
5' RT sr_srg1	TTCATGCCTACCGAGCTCAC		real time PCR primer for *sr_srg1* (*sr16595*) from *S. reilianum*
3' RT sr_srg1	CGACCGAGTTCTGGATGAGC		real time PCR primer for *sr_srg1* (*sr16595*) from *S. reilianum*
5' RT UMAG_02142	GTTCCTTGCCTGGAAAAACG		real time PCR primer for *UMAG_02142*
3' RT UMAG_02142	TGGTGGTCGCTGTACTTTCG		real time PCR primer for *UMAG_02142*
5' RT UHOR_ppi	CATCACCACCGTTGTCACCC		real time PCR primer for *ppi* (*UHOR_05685*) from *U. hordei*
5' RT UHOR_ppi	CTGGAACCTTGGGCCTCGAT		real time PCR primer for *ppi* (*UHOR_05685*) from *U. hordei*
5' RT UHOR_shy1	GCCTACCGTGGTTTGATCCC		real time PCR primer for *UHOR_shy1* (*UHOR_03215)* from *U. hordei*
3' RT UHOR_shy1	TCATTTGGGCTGTGTTTGCG		real time PCR primer for *UHOR_shy1* (*UHOR_03215)* from *U. hordei*
5' RT UHOR_srg1	CGCTGTGTGTCAAGCTGATC		real time PCR primer for *UHOR_srg1* (*UHOR_08490*) from *U. hordei*
3' RT UHOR_srg1	ACCAGTGCGAGTAAGGTTGG		real time PCR primer for *UHOR_srg1* (*UHOR_08490*) from *U. hordei*
5' srg1 prom	ATACCATATGGCGCGGACGAACCGACGACTG	NdeI	forward primer to amplify *srg1* promoter / p123-P_{srg1}mCherry-3xHA cloning
3' srg1 prom	ACATCCATGGTATGTATGTGCGGATGGCAG	NcoI	reverse primer to amplify *srg1* promoter / p123-P_{srg1}mCherry-3xHA cloning
5‘ pScos seq	TGTCCGTGGAATGAACAATG		forward sequencing primer for *U. maydis* cosmid / cosmid complementation assay
3‘ pScos seq	ATCACGAGGCCCTTTCGTC		reverse sequencing primer for *U. maydis* cosmid / cosmid complementation assay
5' UMAG_05966 genomic	ACATGGTACCTTCCACTGCATGAGCGAAAAGGC	KpnI	forward primer to amplify *UMAG_05966* including endogenous promoter and terminator / pUMAG_05966 cloning

Name	Sequence (5’-3’)	Restriction site	Application
3’ UMAG_05966 genomic	TAGTGGCGCGCCAGCCGGTGCGAAGGGCAAGAG	AscI	reverse primer to amplify *UMAG_05966* including endogenous promoter and terminator / pUMAG_05966 cloning
5‘ RB rss1	ATATGCTCTTCACGATATGGGCTTGATGCATGCAACC	SapI	forward primer to amplify right border of *rss1* / *rss1* deletion construct
3’ RB rss1	ATATGCTCTTCTCGCGGCGCGCCAGTTGGGCAACGTCGGCGC	SapI	reverse primer to amplify right border of *rss1* / *rss1* deletion construct
5’ LB rss1	ATATGCTCTTCACAGGGCGCGCCAAATTCCCAAATGGCGCG	SapI	forward primer to amplify left border of *rss1* / *rss1* deletion construct
3’ LB SapI mutagen	ATATGCTCTTCCCTTGCAAGAGCACACGATCCCTGGATCGTG	SapI	reverse primer to mutagenize internal SapI site of left border of *rss1*
5’ LB SapI mutagen	ATATGCTCTTCCAAGATAACGTTACTATAGC	SapI	forward primer to mutagenize internal SapI site of left border of *rss1*
3’ LB rss1	ATATGCTCTTCTGCCGGTGGCGATGCAGCGATTGC	SapI	reverse primer to amplify left border of *rss1* / rss1 deletion construct
5’ LB nested rss1	ACGGGCGAGCGTGGTCTGGTG		forward primer to amplify deletion construct of *rss1*
3’ RB nested rss1	AATCCCGTGGACAGCAGCGTC		reverse primer to amplify deletion construct of *rss1*
5’ rss1	ATATTCATGAGCTCCTCATCCTCCTCGCACAC	BspHI	forward primer to amplify *rss1* / pEntry-rss1 cloning
3' rss1	ATATGCGGCCGCCTAGATGATCAAACGGATAC	NotI	reverse primer to amplify *rss1* / pEntry-rss1, p123-P_{rss1}rss1 cloning
5’ rss1 pro	ATATCATATGAGCCGGTGCGAAGGGCAAGAG	NdeI	forward primer to amplify *rss1* promoter / p123-P_{rss1}rss cloning
5’ pEntry seq	AGTTAGTTACTTAAGCTCG		forward primer for pEntry sequencing
3’ pEntry seq	CAGAGCTGCAGCTGGATGG		reverse primer for pEntry sequencing
5’ RT rss1	ACTCTTCTTCAGGCTCGTTCAAACT		real time PCR primer for *rss1*
3’ RT rss1	CATTCGTGCTTCTAAACGGTAGAG		real time PCR primer for *rss1*

**Name**	**Sequence (5'-3')**	**Restriction site**	**Application**
5' rss1 N-term fusion	ATATGGTCTCTCGCTATGTC ATCCTCATCCTCCTC	BsaI	forward primer to amplify *rss1*-CDS for Golden Gate cloning / p123-P_{rss1}mCherryHA-rss1
3' rss1 N-term fusion	ATATGGCGCGCCTCATAGA TGATCAAACGGATACG	BsaI	reverse primer to amplify *rss1*-CDS for Golden Gate cloning / p123-P_{rss1}mCherryHA-rss1
5' mCherry-BsaI	ATATGGTCTCTATGGTGAGC AAGGGCGAGGAGG	BsaI	forward primer to amplify *mCherry-HA* for Golden Gate cloning / p123-P_{rss1}mCherryHA-rss1
3' mCherry-BsaI	ATATGGTCTCAAGCGCCGC CAGCGCCAGCGTAATCTGG AACATCGTATG	BsaI	reverse primer to amplify *mCherry-HA* for Golden Gate cloning / p123-P_{rss1}mCherryHA-rss1
5' rss1pro GG	ACTCACCCCTCTTCAGCCAA C		forward primer to amplify partial *rss1* promoter for Golden Gate cloning / p123-P_{rss1}mCherryHA-rss1
3' rss1pro GG	ATATGGTCTCACCATGGTG GCGATGCAGCGATTGC	BsaI	reverse primer to amplify partial *rss1* promoter for Golden Gate cloning / p123-P_{rss1}mCherryHA-rss1

* Oligonucleotides were designed with the software CLC Main Workbench (Qiagen, Hilden, Germany) and purchased from Eurofins Genomics (Ebersberg, Germany).

Supplemental Table 4: Plasmids generated and used in this study.

Name	Resistance marker	Cloning and application	Source
p123-P_{srg1}mCherry-3xHA	Amp^R, CBX^R	Plasmid for *U. maydis* UV mutagenesis screen to identify SA sensing and signaling components. The plasmid is based on p123-P_{cmu1}mCherry-3xHA (A. Djamei, unpublished). The *cmu1* promoter is replaced via NdeI-NcoI cloning by 1.5 kb of *UMAG_05967* (*srg1*) promoter. The plasmid can be used for *ip* locus integrations after linearization with SspI.	This study
pUMAG_05966	Amp^R, Hyg^R	Autonomously replicating plasmid for complementation of *U. maydis* UV mutants displaying SA growth defect. *UMAG_05966* including endogenous promoter and terminator sequence (1500 bp and 400 bp, respectively) was introduced into pNEBuH (Weinzierl, 2001) by KpnI-AscI cloning.	This study
pUKOΔrss1	$Spec^R$	Plasmid harboring deletion construct to replace *UMAG_05966* (*rss1*) CDS by Hyg^R cassette. It was generated by SapI-based Golden Gate cloning of the left and right border of *UMAG_05966* CDS and SapI flanked Hyg^R cassette into pUKO-SapI (S. Uhse and A. Djamei, unpublished).	This study
p123-P_{rss1}mCherryHA-rss1	Amp^R, CBX^R	Plasmid encoding UMAG_05966 (Rss1) with N-terminal mCherry-HA fusion. It was used for complementation assays and microscopy studies. The respective construct is under control of *rss1* promoter. It was generated by Golden Gate followed by classical cloning: 200 bp of *rss1* promoter were fused to *mCherry-HA* and *rss1* via BsaI-based Golden Gate reaction. The construct was subsequently cut with HindIII-AscI and inserted into a pre-cut p123-P_{rss1}-rss1 harboring the residual part of the *rss1* promoter. The plasmid can be used for *ip* locus integrations after linearization with SspI.	This study
p123-P_{rss1}rss1	Amp^R, CBX^R	The plasmid harboring *UMAG_05966* (*rss1*) under control of its endogenous promoter was used for complementation of Δrss1 growth defect on SA minimal medium. The plasmid is based on p123 and was cloned via NdeI-NotI. It can be used for ectopic integration into *ip* locus.	This study
pEntry-rss1	Kan^R	pEntry vector containing *rss1* flanked by *att*L sites. *rss1* was amplified and cloned via BspHI-NotI into a NcoI-NotI linearized pEntry4B vector (Thermo Fisher Scientific, Waltham, MA, USA).	This study

Name	Resistance marker	Cloning and application	Source
pGBKT7	Kan^R	Autonomously replicating yeast plasmid suitable for Yeast two-hybrid studies. It contains the Gal4 binding domain (BD) under control of the *ADH* promoter and *TRP1* auxotrophic marker for selection.	Clontech
pCRII-$Rss1_{mut}$	Amp^R, Kan^R	Library of mutagenized *rss1* versions flanked by *att*L-sites. Mutagenesis was achieved by error-prone PCR (JBS Error-Prone PCR-Kit, Jena Bioscience, Jena, Germany) employing pEntry seq primers.	This study
pGBKT7-$Rss1_{1-216}$	Kan^R	Positive control plasmid for transcriptional activation assay. GAL4-BD is fused to Rss1 version that leads to autoactivity. To obtain an autoactive version, *rss1* was mutagenized by error-prone PCR and cloned into pCRII-TOPO (see pCRII-$Rss1_{mut}$). The generated vector library was subjected to Gateway cloning with pGBKT7-GW (A. Djamei, unpublished). The resulting pGBKT7 derivatives were used for yeast transformation. Transformants were screened for the production of autoactive protein fusions on high stringency medium. pGBKT7-$Rss1_{1-216}$ was isolated from yeast, amplified in *E. coli* and sequenced. It encodes a truncated version of Rss1 comprising the N-terminal part of the protein (aa 1-216), which is autoactive.	This study
pGBKT7-rss1	Kan^R	Autonomously replicating yeast vector harboring N-terminal fusion of Gal4 binding domain (BD) with Rss1 under control of *ADH* promoter. The plasmid was generated via Gateway cloning of pEntry-rss1 and pGBKT7-GW (A. Djamei, unpublished).	This study
pGADT7	Amp^R	Autonomously replicating yeast plasmid suitable for Yeast two-hybrid studies. It contains the Gal4 activation domain (AD) under control of the *ADH* promoter and *LEU2* auxotrophic marker for selection.	Clontech
pGADT7-rss1	Amp^R	Autonomously replicating yeast vector harboring N-terminal fusion of Gal4 activation domain (AD) with Rss1 under control of *ADH* promoter. The plasmid was generated via Gateway cloning of pEntry-rss1 and pGADT7-GW (A. Djamei, unpublished).	This study

Supplemental Table 5: *U. maydis* strains used in this study.*

Strain	Genotype	Resistance	Reference
CL13	*a1 bE1 bW2*		Bolker et al. (1995)
CL13Δshy1	*a1 bE1 bW2 ΔUMAG_05230*	Hyg	Rabe et al. (2013)
CL13ΔUMAG_03408	*a1 bE1 bW2 ΔUMAG_03408*	Hyg	Rabe et al. (2013)
CL13Δsrg1	*a1 bE1 bW2 ΔUMAG_05967*	Gent	Rabe et al. (2013)
SG200P$_{srg1}$ mCherry-3xHA	*a1 bE1 bW2 mfa2 ipr[P$_{UMAG_05967}$mCherry-3xHA]ips*	Phleo, CBX	This study
UV1	*a1 bE1 bW2 mfa2 UMAG_05966$_{G629D}$ ipr[P$_{UMAG_05967}$mCherry-3xHA]ips*	Phleo, CBX / UV mutant derived from SG200P$_{srg1}$mCherry-3xHA producing UMAG_05967$_{G629D}$	This study
UV2	*a1 bE1 bW2 mfa2 ΔUMAG_05964-UMAG_05977 ipr[P$_{UMAG_05967}$mCherry-3xHA]ips*	Phleo, CBX / UV mutant derived from SG200P$_{srg1}$mCherry-3xHA that lost more than 45 kbp from chr20	This study
UV3	*a1 bE1 bW2 mfa2 ΔUMAG_05964-UMAG_05977 ipr[P$_{UMAG_05967}$mCherry-3xHA]ips*	Phleo, CBX / UV mutant derived from SG200P$_{srg1}$mCherry-3xHA that lost more than 45 kbp from chr20	This study
UV4	*a1 bE1 bW2 mfa2 ΔUMAG_05964-UMAG_05977 ipr[P$_{UMAG_05967}$mCherry-3xHA]ips*	Phleo, CBX / UV mutant derived from SG200P$_{srg1}$mCherry-3xHA that lost more than 45 kbp from chr20	This study
UV5	*a1 bE1 bW2 mfa2 ΔUMAG_05964-UMAG_05977 ipr[P$_{UMAG_05967}$mCherry-3xHA]ips*	Phleo, CBX / UV mutant derived from SG200P$_{srg1}$mCherry-3xHA that lost more than 45 kbp from chr20	This study
UV6	*a1 bE1 bW2 mfa2 UMAG_05966$_{F303S}$ ipr[P$_{UMAG_05967}$mCherry-3xHA]ips*	Phleo, CBX / UV mutant derived from SG200P$_{srg1}$mCherry-3xHA producing UMAG_05967$_{F303S}$	This study
UV7	*a1 bE1 bW2 mfa2 ΔUMAG_05964-UMAG_05977 ipr[P$_{UMAG_05967}$mCherry-3xHA]ips*	Phleo, CBX / UV mutant derived from SG200P$_{srg1}$mCherry-3xHA that lost more than 45 kbp from chr20	This study

Strain	Genotype	Resistance	Reference
UV1/pUMAG_ 05966	*a1 bE1 bW2 mfa2 UMAG_05966*$_{G629D}$ *ipr[P*$_{UMAG\_05967}$*mCherry-3xHA]ips* pUMAG_05966	Phleo, CBX, Hyg	This study
UV2/pUMAG_ 05966	*a1 bE1 bW2 mfa2 ΔUMAG_05964-UMAG_05977 ipr[P*$_{UMAG\_05967}$*mCherry-3xHA]ips* pUMAG_05966	Phleo, CBX, Hyg	This study
UV3/pUMAG_ 05966	*a1 bE1 bW2 mfa2 ΔUMAG_05964-UMAG_05977 ipr[P*$_{UMAG\_05967}$*mCherry-3xHA]ips* pUMAG_05966	Phleo, CBX, Hyg	This study
UV4/pUMAG_ 05966	*a1 bE1 bW2 mfa2 ΔUMAG_05964-UMAG_05977 ipr[P*$_{UMAG\_05967}$*mCherry-3xHA]ips* pUMAG_05966	Phleo, CBX, Hyg	This study
UV5/pUMAG_ 05966	*a1 bE1 bW2 mfa2 ΔUMAG_05964-UMAG_05977 ipr[P*$_{UMAG\_05967}$*mCherry-3xHA]ips* pUMAG_05966	Phleo, CBX, Hyg	This study
UV6/pUMAG_ 05966	*a1 bE1 bW2 mfa2 UMAG_05966*$_{F303S}$ *ipr[P*$_{UMAG\_05967}$*mCherry-3xHA]ips* pUMAG_05966	Phleo, CBX, Hyg	This study
UV7/pUMAG_ 05966	*a1 bE1 bW2 mfa2 ΔUMAG_05964-UMAG_05977 ipr[P*$_{UMAG\_05967}$*mCherry-3xHA]ips* pUMAG_05966	Phleo, CBX, Hyg	This study
CL13Δrss1	*a1 bE1 bW2 ΔUMAG_05966*	Hyg	This study
CL13Δrss1-mCherryHA-rss1	*a1 bE1 bW2 ΔUMAG_05966 ipr[P*$_{UMAG\_05966}$*mCherryHA-UMAG_05966]ips*	Hyg, CBX	This study
CL13Δrss1-rss1	*a1 bE1 bW2 ΔUMAG_05966 ipr[P*$_{UMAG\_05966}$*UMAG_05966]ips*	Hyg, CBX	This study
SG200	*a1 mfa2 bE1 bW2*	Phleo	Kamper et al. (2006)
SG200Δrss1	*a1 mfa2 bE1 bW2 ΔUMAG_05966*	Phleo, Hyg	This study

* Plasmids were integrated into the *ip* locus and verified by Southern and/or PCR. Strain deletions were verified by Southern analysis. The nomenclature for *ip*-locus integrations was adopted from Brachmann (2001). Abbreviations: CBX = Carboxin, Gent = Geneticin G418, Hyg = Hygromycin, Phleo = Phleomycin.

Supplemental Table 6: *S. cerevisiae* strains used in this study.*

Strain	Genotype	Resistance/ Application	Reference
AH109	*MATa, trp1-901, leu2-3, 112, ura3-52, his3-200, gal4Δ, gal80Δ, LYS2 : : GAL1*$_{UAS}$*-GAL1*$_{TATA}$*-HIS3, MEL1, GAL2*$_{UAS}$*-GAL2*$_{TATA}$*-ADE2, URA3 : : MEL1*$_{UAS}$*-MEL1*$_{TATA}$*-lacZ*	Transcriptional activation assay	Clontech
AH109-BD	*MATa, trp1-901, leu2-3, 112, ura3-52, his3-200, gal4Δ, gal80Δ, LYS2 : : GAL1*$_{UAS}$*-GAL1*$_{TATA}$*-HIS3, MEL1, GAL2*$_{UAS}$*-GAL2*$_{TATA}$*-ADE2, URA3 : : MEL1*$_{UAS}$*-MEL1*$_{TATA}$*-lacZ,* pGBKT7	Transcriptional activation assay / Homodimerization assay	This study
AH109-BD-Rss1$_{1-216}$	*MATa, trp1-901, leu2-3, 112, ura3-52, his3-200, gal4Δ, gal80Δ, LYS2 : : GAL1*$_{UAS}$*-GAL1*$_{TATA}$*-HIS3, MEL1, GAL2*$_{UAS}$*-GAL2*$_{TATA}$*-ADE2, URA3 : : MEL1*$_{UAS}$*-MEL1*$_{TATA}$*-lacZ,* pGBKT7-Rss1$_{1-216}$	Transcriptional activation assay / Homodimerization assay	This study
AH109-BD-Rss1	*MATa, trp1-901, leu2-3, 112, ura3-52, his3-200, gal4Δ, gal80Δ, LYS2 : : GAL1*$_{UAS}$*-GAL1*$_{TATA}$*-HIS3, MEL1, GAL2*$_{UAS}$*-GAL2*$_{TATA}$*-ADE2, URA3 : : MEL1*$_{UAS}$*-MEL1*$_{TATA}$*-lacZ*, pGBKT7-rss1	Transcriptional activation assay / Homodimerization assay	This study
Y187	*MAT*α*, ura3-52, his3-200, ade2-101, trp1-901, leu2-3, 112, gal4Δ, gal80Δ, met*$^{-}$*, URA3 : : GAL1*$_{UAS}$*–Gal1*$_{TATA}$*–LacZ*	Homodimerization assay	Clontech
Y187-AD	*MAT*α*, ura3-52, his3-200, ade2-101, trp1-901, leu2-3, 112, gal4Δ, gal80Δ, met–, URA3 : : GAL1*$_{UAS}$*–Gal1*$_{TATA}$*–LacZ,* pGADT7	Homodimerization assay	This study
Y187-AD-Rss1	*MAT*α*, ura3-52, his3-200, ade2-101, trp1-901, leu2-3, 112, gal4Δ, gal80Δ, met–, URA3 : : GAL1*$_{UAS}$*–Gal1*$_{TATA}$*–LacZ,* pGADT7-rss1	Homodimerization assay	This study

*After transformation with pGADT7 and pGBKT7 derivatives, strains were verified by PCR.

References

Bolker, M., et al., 1995. Genetic regulation of mating and dimorphism in Ustilago maydis. Can. J. Bot. 73, 329-342.

Brachmann, A., Die frühe Infektionsphase von Ustilago maydis: Genregulation durch das bE/bW-Heterodimer. Ludwig-Maximilians-Universität München, 2001.

Flor-Parra, I., M. Vranes, et al. (2006). "Biz1, a zinc finger protein required for plant invasion by Ustilago maydis, regulates the levels of a mitotic cyclin." Plant Cell 18(9): 2369-2387.

Kamper, J., et al., 2006. Insights from the genome of the biotrophic fungal plant pathogen Ustilago maydis. Nature. 444, 97-101.

Rabe, F., et al., 2013. Degradation of the plant defence hormone salicylic acid by the biotrophic fungus Ustilago maydis. Mol Microbiol. 89, 179-88.

Weinzierl, G., Isolierung und Charakterisierung von Komponenten der b-vermittelten Regulationskaskade in Ustilago maydis. Philipps-Universität Marburg, 2001.
